# A new family of diprotodontian marsupials from the latest Oligocene of Australia and the evolution of wombats, koalas, and their relatives (Vombatiformes)

**DOI:** 10.1038/s41598-020-66425-8

**Published:** 2020-06-25

**Authors:** Robin M. D. Beck, Julien Louys, Philippa Brewer, Michael Archer, Karen H. Black, Richard H. Tedford

**Affiliations:** 1 Ecosystems and Environment Research Centre, School of Science, Engineering and Environment, University of Salford, Manchester, UK; 2 PANGEA Research Centre, School of Biological, Earth and Environmental Sciences, University of New South Wales, Sydney, New South Wales Australia; 3 Australian Research Centre for Human Evolution, Environmental Futures Research Institute, Griffith University, Queensland, Australia; 4 Department of Earth Sciences, Natural History Museum, London, United Kingdom; 5 Division of Paleontology, American Museum of Natural History, New York, USA

**Keywords:** Palaeontology, Phylogenetics, Taxonomy

## Abstract

We describe the partial cranium and skeleton of a new diprotodontian marsupial from the late Oligocene (~26–25 Ma) Namba Formation of South Australia. This is one of the oldest Australian marsupial fossils known from an associated skeleton and it reveals previously unsuspected morphological diversity within Vombatiformes, the clade that includes wombats (Vombatidae), koalas (Phascolarctidae) and several extinct families. Several aspects of the skull and teeth of the new taxon, which we refer to a new family, are intermediate between members of the fossil family Wynyardiidae and wombats. Its postcranial skeleton exhibits features associated with scratch-digging, but it is unlikely to have been a true burrower. Body mass estimates based on postcranial dimensions range between 143 and 171 kg, suggesting that it was ~5 times larger than living wombats. Phylogenetic analysis based on 79 craniodental and 20 postcranial characters places the new taxon as sister to vombatids, with which it forms the superfamily Vombatoidea as defined here. It suggests that the highly derived vombatids evolved from wynyardiid-like ancestors, and that scratch-digging adaptations evolved in vombatoids prior to the appearance of the ever-growing (hypselodont) molars that are a characteristic feature of all post-Miocene vombatids. Ancestral state reconstructions on our preferred phylogeny suggest that bunolophodont molars are plesiomorphic for vombatiforms, with full lophodonty (characteristic of diprotodontoids) evolving from a selenodont morphology that was retained by phascolarctids and ilariids, and wynyardiids and vombatoids retaining an intermediate selenolophodont condition. There appear to have been at least six independent acquisitions of very large (>100 kg) body size within Vombatiformes, several having already occurred by the late Oligocene.

## Introduction

The three living species of wombat (*Vombatus ursinus*, *Lasiorhinus latifrons* and *L. krefftii*; family Vombatidae) and the koala (*Phascolarctos cinereus*; family Phascolarctidae) are among the most iconic and unusual of Australia’s marsupials. They are the only survivors of the clade Vombatiformes, one of the two major subclades within the order Diprotodontia^1,2^; the other, Phalangerida, includes possums, kangaroos, wallabies and rat kangaroos. Fossil vombatiforms were highly diverse and included the large-bodied, herbivorous diprotodontids, the possibly tapir- or chalicothere-like palorchestids, and the carnivorous thylacoleonid “marsupial lions”^3–6^. Vombatiformes suffered extensive extinctions during the Pleistocene^7,8^: the last surviving members of several families (Diprotodontidae, Palorchestidae and Thylacoleonidae) went extinct, as did several vombatid and phascolarctid species, leaving the modern wombats and koala as the only remnants of this former diversity.

Monophyly of Vombatiformes is relatively uncontroversial^1,2,9,10^, but relationships within the clade remain unclear^3,4,6,9,11^. In part, this is because the best known forms are also the youngest and most derived. They include the rhino-sized (>2 tonne) diprotodontid *Diprotodon optatum*, the bizarre palorchestid *Palorchestes azael*, and the hypercarnivorous thylacoleonid *Thylacoleo carnifex*, all of which are known from relatively complete specimens from the Pleistocene^3–6,12–14^. Among modern forms, the koala exhibits extreme modifications of its masticatory and auditory anatomy (connected with its diet of eucalyptus and its sedentary lifestyle, respectively^15,16^), while the skull and skeleton of wombats is also highly specialized^16,17^. Another major difficulty in unravelling vombatiform relationships is the fact that the premolars and molars of modern wombats are hypselodont (ever-growing) and their unworn occlusal morphology – a fundamental source of information for mammalian phylogeny - is lost through wear early in post-natal life^17^.

The discovery of well-preserved remains of more plesiomorphic vombatiforms from Oligo-Miocene fossil sites in Australia has given additional insight into the evolutionary history of the clade^3,4^. However, a number of key questions remain unresolved, particularly regarding the origin and interfamilial relationships of vombatids^18–21^. Here we describe a cranium and associated partial skeleton of a new, highly distinctive fossil vombatiform from the late Oligocene (~26-25 Ma old) Pinpa Local Fauna of the Namba Formation, in the Lake Eyre Basin of northeastern South Australia. This taxon is one of the oldest Australian marsupials known from an associated skeleton. It provides key new data for understanding the evolutionary history of vombatiforms, including the evolution of digging adaptations, homologies of molar structures, phylogenetic relationships, and patterns of dental and body mass evolution within the clade.

### Systematic palaeontology

Order Diprotodontia Owen, 1866 New Definition (see Table 1)

**Table 1:** Proposed phylogenetic definitions (all stem-based) for selected clades.

Clade	Phylogenetic Definition
Diprotodontia	The most inclusive clade including *Phalanger orientalis* but not *Dasyurus viverrinus*, *Dromiciops gliroides*, *Notoryctes typhlops* or *Perameles nasuta*
Vombatiformes	the most inclusive clade including *Vombatus ursinus* but not *Phalanger orientalis*
Vombatomorphia	the most inclusive clade including *Vombatus ursinus* but not *Phascolarctos cinereus*
Phascolarctomorphia	the most inclusive clade including *Phascolarctos cinereus* but not *Vombatus ursinus*
Vombatoidea	the most inclusive clade including *Vombatus ursinus* but not *Diprotodon opatum*, *Phascolarctos cinereus* or *Thylacoleo carnifex*
Diprotodontoidea	the most inclusive clade including *Diprotodon opatum*, but not *Phascolarctos cinereus*, *Thylacoleo carnifex* or *Vombatus ursinus*

Suborder Vombatiformes Woodburne, 1984 New Definition (see Table 1)

Infraorder Vombatomorphia Aplin and Archer, 1987 New Definition (see Table 1)

Superfamily Vombatoidea Kirsch, 1968 New Definition (see Table 1)

Family Mukupirnidae

Type genus: *Mukupirna*

Included taxa: *Mukupirna nambensis* new species

Generic diagnosis: As for the only known species

### Type species

*Mukupirna nambensis* gen. et. sp. nov.

Differential diagnosis: differs from known members of Wynyardiidae in possessing a P3 that lacks a posterolingual cusp (=”hypocone”), less well-developed selenodonty, a less well-developed masseteric process, palatal vacuities entirely enclosed by the palatines, a proportionately longer deltopectoral crest and broader distal end of the humerus (Epicondylar index = 0.44^22^), a proportionately longer olecranon of the ulna (Index of Fossorial Ability = 0.42^23^), and a much larger body size (estimated body mass based on postcranial measurements = 143–171 kg); differs from vombatids in lacking bilobate molars (molars are only slightly bilobate in *Nimbavombatus*, *Rhizophascolonus* and *Warendja*, but strongly bilobate in other vombatids); differs from all vombatids except *Nimbavombatus* in retaining three upper incisors and the upper canine; differs from *Nimbavombatus* in larger size, more bicuspid P3, and palatal vacuities entirely enclosed by the palatines; differs from vombatids known from postcranial remains in lacking a laterally extensive deltopectoral crest of the humerus; differs from hypselodont vombatids in having closed premolar and molar roots; differs from known members of Thylacoleonidae in retaining only a single upper premolar (P3), with this tooth not as elongate or bladelike, lacking a marked reduction in molar size posteriorly, having a proportionately longer deltopectoral crest and broader distal end of the humerus, and having a proportionately longer olecranon of the ulna; differs from known members of Phascolarctidae in lacking strongly selenodont molars, having a less well-developed masseteric process, a proportionately longer deltopectoral crest and broader distal end of the humerus, and a proportionately longer olecranon of the ulna; differs from known members of Ilariidae in lacking posterobuccal and lingual cusps on P3, in lacking strongly selenodont molars, and in lacking a well-developed masseteric process; differs from known members of Diprotodontidae and Palorchestidae in lacking a molariform P3, molars not strongly bilophodont, in lacking a well-developed masseteric process, and in retaining palatal vacuities. *Mukupirna nambensis* cannot be compared directly with *Marada arcanum* (the only known representative of the vombatiform family Maradidae), because *Mu. nambensis* is only known from the cranium and upper dentition whereas *Ma. arcanum i*s known only from the lower dentition, and it is possible that they represent the same taxon or are closely related (see the supplementary information).

### Holotype

AMNH FM 102646 (previously, QMAM 168^24^), a badly crushed cranium (preserved length = 197 mm; dorsal surface not preserved) with left and right P3-M4, and associated partial postcranial skeleton comprising vertebrae, ribs, left and right scapulae, left humerus, left ulna, left radius, left and right femora, left tibia, left fibula, and parts of the autopodia. The adult dentition is fully erupted, except possibly for M4, which does not appear to be in line with the occlusal surfaces of M1-3 (although this may be the result of post-mortem displacement); the molars are only lightly worn. In the postcranium, most fracturing has occurred at the epiphyseal plates. Collectively this suggests that this individual was probably a late subadult or young adult.

### Etymology

The generic name is from the words *muku* (“bones”) and *pirna* (“big”) in the Dieri (Diyari) language traditionally spoken in the area around Lake Eyre and refers to the large size of the animal. The species name *nambensis* is after the Namba Formation in which the only known specimen was found.

### Type Locality and Age

Lake Pinpa Site C, Namba Formation, Lake Frome area, South Australia. The Namba Formation has been correlated with the Etadunna Formation, which has been estimated to be 26-24 Ma old (i.e. latest Oligocene) on the basis of isotopic, foraminiferal, magnetostratigraphic and radiometric (Rb-Sr dating of illite) data^25^. More recently, the Etadunna Formation has been proposed to be 26.1-23.6 Ma old based on a best-fit age-model of magnetostratigraphic data^26^. The Pinpa Local Fauna is the oldest of the three distinct faunal units recovered from stratigraphic levels in the Namba Formation, and has been correlated with the oldest faunal zone (Zone A) of the Etadunna Formation, which has been dated as 25.3-24.9 Ma old (chrons 7An and 7Ar) based on magnetostratigraphy^25^. In summary, available evidence suggests a probable age of between approximately 26 and 25 MYA for the Pinpa Local Fauna.

### Comparisons between *Mukupirna nambensis* and other vombatiforms

**Figure 1:**
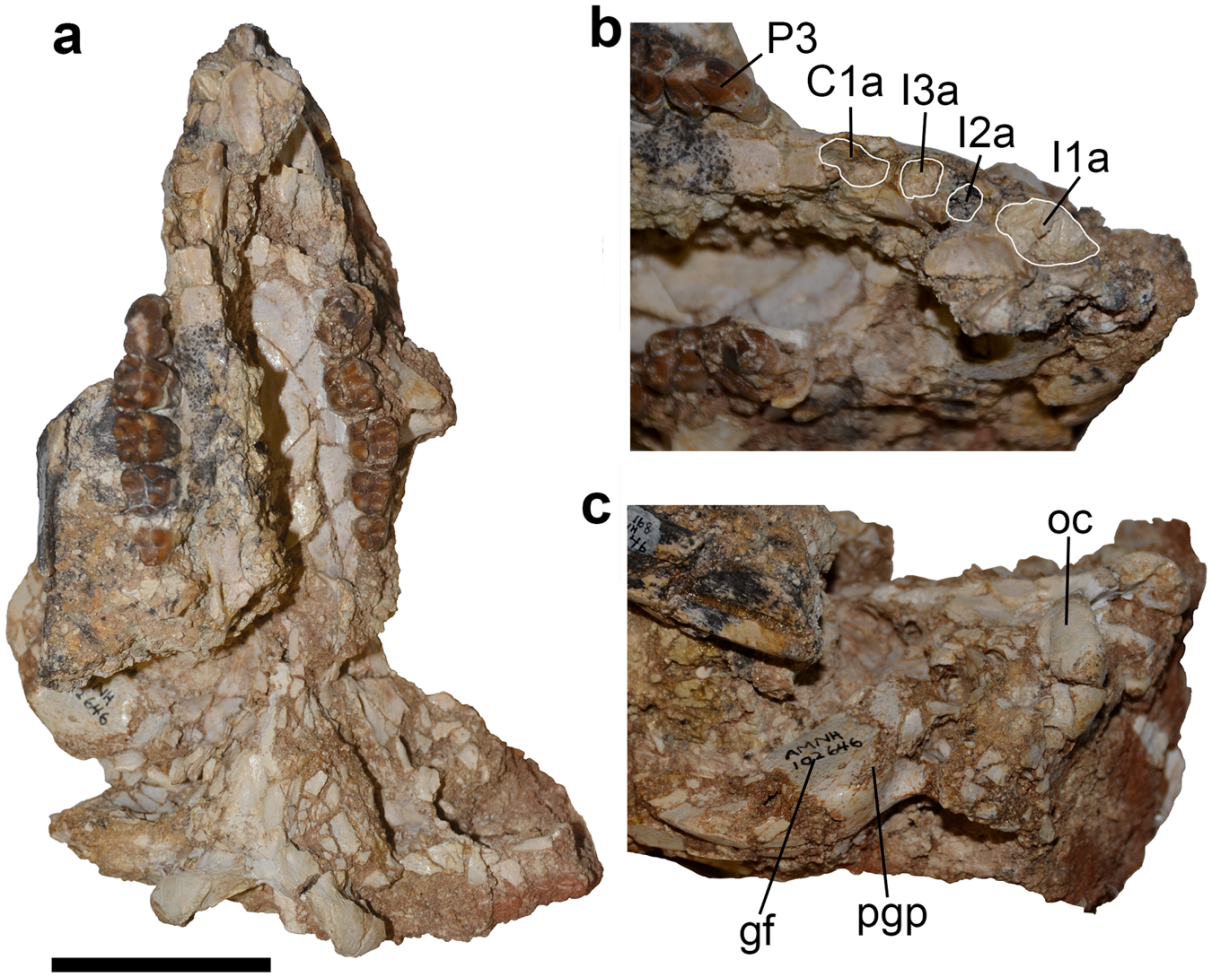
Cranium of holotype and only known specimen of *Mukupirna nambensis* gen. et. sp. nov. (AMNH FM 102646). (**a**) Cranium in ventral view, (**b**) rostral region of right side of cranium in ventromedial view, (**c**) posterior region of right side of cranium in ventromedial view. I1a, alveolus for first upper incisor; I2a, alveolus for second upper incisor; I3a, alveolus for third upper incisor; C1a, alveolus for upper canine; gf, glenoid fossa; oc, occipital condyle; P3, third upper premolar; pgp, postglenoid process. Scale bar = 5 cm.

**Figure 2:**
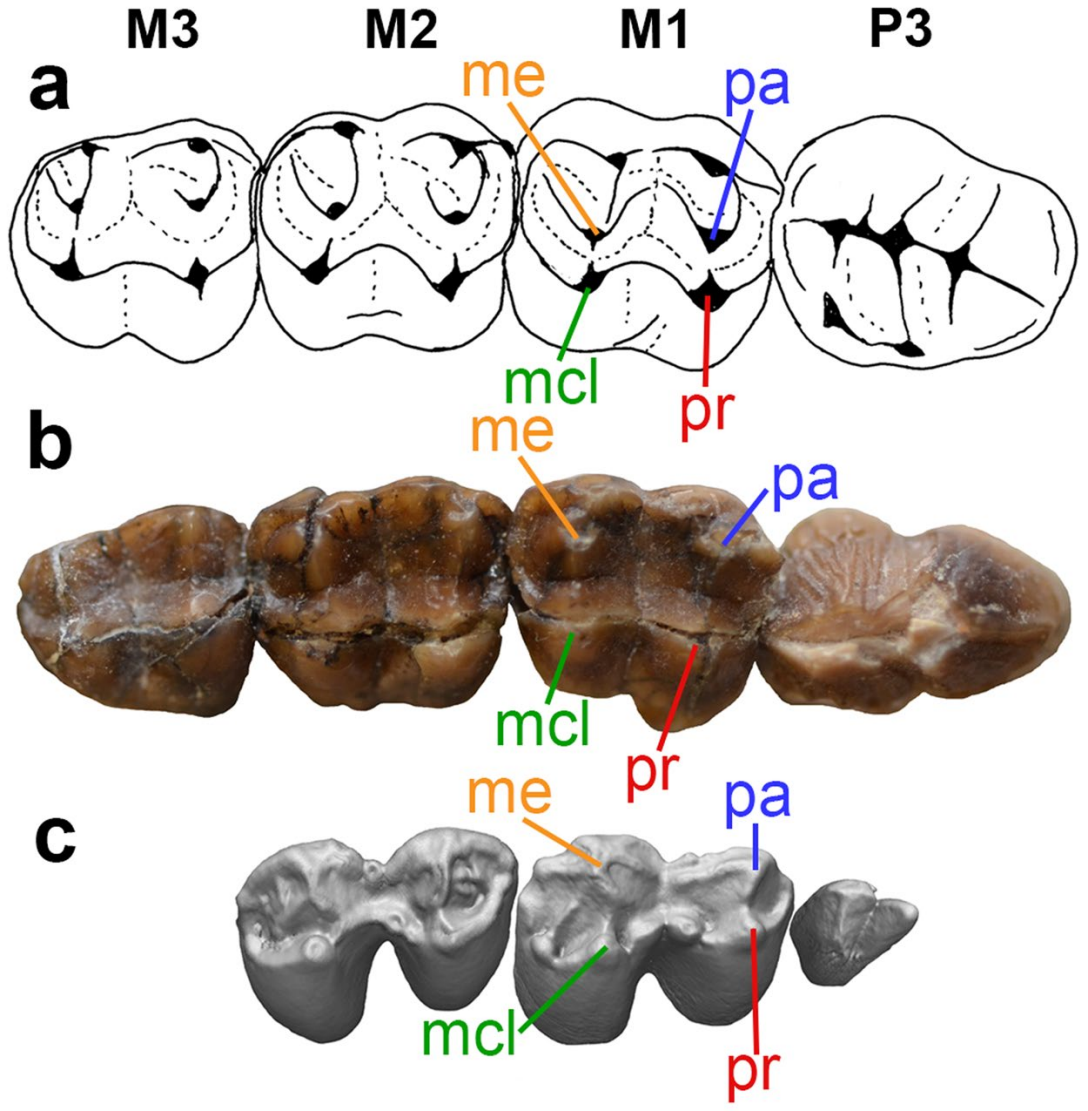
Upper right postcanine dentitions of selected vombatiforms. (**a**) Line drawing of left P3 M1-3 (reversed) of the wynyardiid *Muramura pinpensis* (holotype SAM P36160 and paratype SAM P36161), modified from Pledge (2003: fig. 19.2), (**b**) photograph of right P3 M1-3 of *Mukupirna nambensis* (holotype AMNH FM 102646), (**c**) CT reconstruction of unworn left P3 M1-2 of extant vombatid *Vombatus ursinus* (NMV C19028). M1, first upper molar; M2, second upper molar; M3, third upper molar; mcl, metaconular hypocone; me, metacone; P3, third upper premolar; pa, paracone; pr, protocone.

The preserved craniodental morphology of *Mukupirna* (Figs. 1, 2) appears to be approximately intermediate between that of wynyardiids such as *Namilamadeta* and *Muramura* on the one hand, and that of definitive vombatids on the other^16,17,27–30^. Based on the shapes of the preserved alveoli (Fig. 1b), the upper first incisor of *Mukupirna* was proportionately larger than those of *Namilamadeta* and *Muramura*, but the second and third incisors were still present. In vombatids, I1 is very large and (with the probable exception of *Nimbavombatus*^20^) is the only upper incisor present^17^. Also based on alveolar evidence (Fig. 1b), *Mukupirna* retained a large, single-rooted upper canine, which is a plesiomorphic feature seen in wynyardiids and several other vombatiforms, including phascolarctids, ilariids, thylacoleonids and some diprotodontoids^27–30^. However, an upper canine is absent in all vombatids described to date except *Nimbavombatus*^20^.

Although it is somewhat worn or damaged apically, the distinctly bicuspid P3 of *Mukupirna* (Figs. 1b, 2b) clearly differs from the more strongly bladed P3 of phascolarctids, thylacoleonids and wynyardiids (Fig. 2a), and from the relatively molariform P3 of ilariids, diprotodontids and palorchestids^3,5,9^. The P3 of *Mukupirna* shows vertical ridging or fluting (most obvious on its posterior half; Fig. 2b); among other vombatiforms, somewhat similar vertical ridging is seen in the P3 of wynyardiids^29,30^, but also phascolarctids^31,32^. The P3 of *Mukupirna* also lacks any lingual cusps (Figs. 1b, 2b), unlike the condition seen in most vombatiforms besides thylacoleonids^9^.

Interestingly, the unworn P3 of the extant common wombat *Vombatus ursinus* (assuming that this is indeed P3 and not a retained dP3) is also bicuspid and without evidence of lingual cusps (Fig. 2c), although it differs in being proportionately much smaller and more distinctly bicuspid, and in lacking vertical ridging. However, the P3 (again, if it is not dP3) of *Lasiorhinus latifrons* and of fossil vombatids is sub-triangular and not bicuspid, and a small posterolingual cusp is typically present^20,21,33,34^. The P3 of vombatids is also usually smaller than the anterior upper molars, in contrast to *Mukupirna* (Fig. 2b,c)^20,21,33,34^. Thus, the bicuspid P3 of *Mukupirna* and *Vombatus* may be the result of homoplasy; overall, the P3 of *Mukupirna* most closely resembles that of the wynyardiid *Namilamadeta* (Fig. 2b)^28,30^. However, the enamel on the labial surface of P3 of *Mukupirna* extends onto the root of the tooth, which is a distinctive feature of most vombatids but apparently absent in other vombatiforms, including wynyardiids.

The molar morphology of *Mukupirna* (Fig. 2b) is very similar to that of the wynyardiids *Namilamadeta* and *Muramura* (Fig. 2a) in being selenolophodont, i.e., exhibiting both lophodont and selenodont features^27–30^. Prominent stylar cusps are present along the labial margin of the molars, with the paracone and metacone located further lingually (Fig. 2b), but they are not as centrally placed on the tooth crown as they are in wynyardiids (Fig. 2a). A weak selenodont pattern is apparent in *Mukupirna*, at least on M1-2, with identifiable pre- and postparacristae and pre- and postmetacristae extending from the paracone and metacone respectively (Fig. 2b); these crests are also present, but much better developed, in wynyardiids (Fig. 2a). Weak lophs connect the paracone to the protocone and the metacone to the metaconular hypocone in *Mukupirna* (Fig. 2b), again as in wynyardiids (Fig. 2a). The upper molars of most other vombatiforms are either fully lophodont (diprotodontids, palorchestids), fully selenodont (ilariids, phascolarctids), or bunodont-bunolophodont (thylacoleonids)^3,5,9^. Intriguingly, however, the same basic occlusal morphology seen in *Mukupirna* occurs in unworn upper molars of the plesiomorphic fossil vombatids *Nimbavombatus* and *Rhizophascolonus* and juveniles of the living wombats *Vombatus* and *Lasiorhinus*^20,21^, in which homologues of the paracone and metacone retain traces of a selenodont pattern, but are also connected by weak lophs to the protocone and metaconular hypocone respectively (Fig. 2c).

In contrast to wynyardiids and most other vombatiforms (in which a distinct cervix usually separates the root and crown), there is no clear distinction between the root and crown of the upper molars of *Mukupirna* (Fig. 1a).This is another feature also seen in vombatids, although *Mukupirna* differs from vombatids in that the enamel of its molars does not extend down the lingual surface of the roots. The molar roots of *Mukupirna* are long, and the lingual roots extend ventrally far beyond the molar alveoli, whereas the labial roots are not visible, again as in vombatids. However, *Mukupirna* has closed molar roots, and so in this respect is unlike all known vombatids except the early Miocene *Nimbavombatus* and *Rhizophascolonus*^17,21,35,36^. The occlusal surface of early Miocene vombatids was subject to moderate to severe amounts of wear (particularly towards the anterior end of the molar row). The most extreme wear is seen in *R. crowcrofti* where the occlusal morphology appears to have been obliterated relatively early in the animal’s life, leaving an enamel perimeter surrounding a dentine wear surface^35,36^, as is the case in hypselodont vombatids. By contrast, the molars of *Mukupirna* evidently retained their original occlusal morphology into at least early adulthood, with no evidence of accelerated wear in the M1 position.

Although the anterior part of the zygomatic arch is poorly preserved in AMNH FM 102646, the masseteric process appears to be weakly developed or absent (Fig. 1a), as it is in vombatids^17^; by contrast, this process is distinct in most other vombatiforms, including wynyardiids^3,5,27–30^, although it is also absent in most thylacoleonids^37,38^. In most vombatids (with the notable exceptions of *Warendja* and *Nimbavombatus*^17,34^), a very large fossa extends across the lateral surface of the maxilla and jugal, at the anterior end of the zygomatic arch; in extant wombats, this fossa has been shown to house a greatly enlarged superficial masseter^17,39^, which generates high occlusal forces during the medially-directed power stroke of the lower jaw^17,39,40^. The wynyardiids *Muramura* and *Namilamadeta* have a similarly-positioned but much smaller fossa^27,28]^, and the preserved part of the jugal of *Mukupirna* also preserves a shallow but distinct fossa on its lateral surface. The presence of this fossa in wynyardiids and *Mukupirna* might represent a precursor of the much larger fossa seen in most vombatids, in which case, its absence in *Warendja* and *Nimbavombatus* is presumably secondary; alternatively, it may instead reflect the presence of enlarged snout musculature in wynyardiids and *Mukupirna*^1^. Palatal vacuities are present in *Mukupirna* and appear to be entirely enclosed by the palatine bones, as in most vombatids and also the modern koala *Phascolarctos cinereus*. In wynyardiids, thylacoleonids, fossil phascolarctids and the vombatid *Nimbavombatus*, these vacuities are between the maxillae and palatines, which is probably the plesiomorphic condition within Marsupialia^41^. Diprotodontids and palorchestids lack palatal vacuities^3,5^.

The glenoid fossa of *Mukupirna* is planar, and the postglenoid process also appears to have been either very weakly developed or entirely absent, although the region may be damaged in AMNH FM 102646 (Fig. 1c). By contrast, in most vombatiforms (including wynyardiids), the glenoid fossa has a raised articular eminence anteriorly and distinct mandibular fossa posteriorly, and the postglenoid process is well-developed^3,5,16^. The overall morphology of the glenoid region of *Mukupirna* somewhat resembles that of the plesiomorphic vombatid *Warendja wakefieldi*, which is also planar with a very weakly developed postglenoid process^17,34^. The glenoid region of other vombatids known from cranial remains is highly specialised, with a mediolaterally broad and convex glenoid fossa that lacks any trace of a postglenoid process^16^. The auditory region of AMNH FM 102646 is also damaged, but it appears that the zygomatic epitympanic sinus of the squamosal was either absent or very shallow (Fig. 1c); vombatids are also characterised by a shallow and laterally open zygomatic epitympanic sinus, in contrast to most other diprotodontians in which this sinus is largely enclosed and invades deep into the zygomatic process of the squamosal^16^.

**Figure 3:**
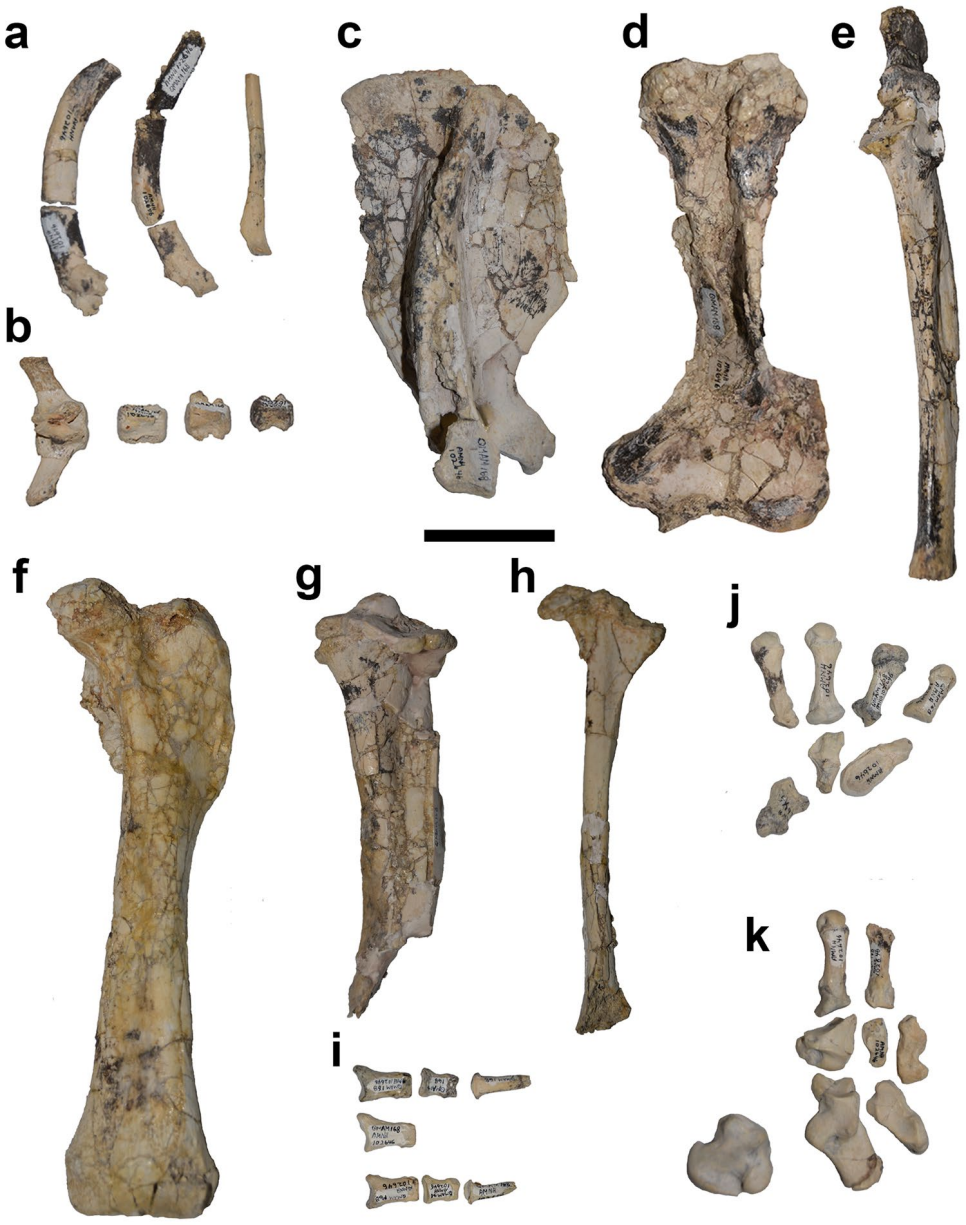
Selected postcranial elements of holotype of *Mukupirna nambensis* gen. et. sp. nov. (AMNH FM 102646). (**a**) ribs, (**b**) caudal vertebrae, (**c**) right scapula, (**d**) left humerus, (**e**) left ulna, (**f**) left femur, (**g**) left tibia, (**h**) left fibula, (**i**) phalanges, (**j**) left carpals and metacarpals, (**k**) left tarsals and metatarsals. Scale bar = 5 cm.

**Figure 4:**
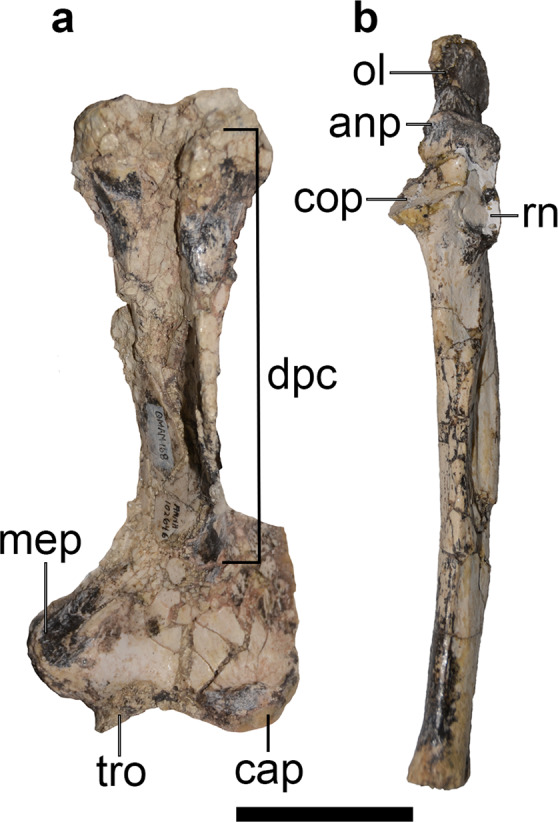
Selected forelimb elements of holotype of *Mukupirna nambensis* gen. et. sp. nov. (AMNH FM 102646). (**a**) left humerus, (**b**) left ulna. anp, anconeal process; cap, capitulum; cop, coronoid process; dpc, deltopectoral crest; mep, medial epicondyle; ol, olecranon; rn, radial notch; tro, trochlea. Scale bar = 5 cm.

The postcranium of *Mukupirna* (Figs. 3, 4) exhibits a number of features that are indicative of digging behaviour^42,43^. The humerus is broad distally, giving an Epicondylar Index (= humeral epicondylar width/humeral length)^23^ of 0.44, similar to that of modern wombat species *Lasiorhinus latifrons* and *Vombatus ursinus* (Fig. 4a and Table 1). The olecranon process of the ulna is elongate in *Mukupirna*, providing increased mechanical advantage to the triceps brachii when extending the forearm, which again is common in digging mammals. The Index of Fossorial Ability (=olecranon length/[total ulnar length-olecranon length]) of *Mukupirna* s 0.42, which is similar to *Vombatus ursinus* but somewhat less than in *Lasiorhinus latifrons* (Fig. 4b and Table 2). A distinct third trochanter is present on the femur, which is unusual among marsupials, and which indicates the presence of well-developed gluteal musculature that may be connected with digging behaviour^44,45^. Most of these features are absent or much less well-developed in other vombatiforms (including wynyardiids), but they are present in vombatids^17,46,47^. The wide distal end of the humerus of *Mukupirna* and vombatids is largely due to a prominent medial epicondyle, which reflects the presence of powerful extensors and pronators of the forearm^17,42,43^. The manual and pedal phalanges of *Mukupirna* are strikingly similar to those of vombatids and also the ilariid *Ilaria*^46^, and are strongly indicative of digging behaviour: they are dorsoventrally flattened (especially the unguals), and their distal ends taper dorsoventrally. In addition, manual phalanx V of *Mukupirna* exhibits a medial twist at its distal end, and lateral buttressing of its proximal end, which is a distinctive feature also seen in vombatids; this morphology serves to position digit V close to digits I-IV, and in living wombats allows the manus to be used as a shovel during digging.

**Table 2:** Epicondylar Index (=humeral epicondylar width/humeral length) and Index of Fossorial Ability (=olecranon length/(total ulnar length-olecranon length) for *Mukupirna nambensis* and selected other vombatiforms and other marsupials, plus ancestral state reconstructions for Vombatiformes and selected vombatiform subclades (see Table 1 and Figs. 5, 6).

Taxon	EI	IFA
*Mukupirna nambensis*	0.44	0.42
*Lasiorhinus latifrons*	0.44	0.58
*Phascolonus gigas*	0.53	0.73
*Vombatus ursinus*	0.45	0.42
Vombatoidea (ASR)	0.43 (0.37–0.48)	0.41 (0.31–0.50)
*Diprotodon opatum*	0.35	0.19
*Kolopsis torus*	0.31	N/A
*Neohelos stirtoni*	0.37	0.28
*Ngapakaldia* spp.	0.38	0.40
*Nimbadon lavarackorum*	0.34	0.23
*Palorchestes azael*	0.61	0.46
*Palorchestes painei*	0.56	N/A
*Propalorchestes novaculacephalus*	0.43	N/A
*Zygomaturus trilobus*	N/A	0.18
Diprotodontoidea (ASR)	0.36 (0.32–0.42)	0.33 (0.23–0.42)
*Ilaria illumidens*	N/A	0.31^a^
*Muramura williamsi*	0.33	0.29
Vombatomorphia (ASR)	0.35 (0.29–0.42)	0.31 (0.18–0.44)
*Phascolarctos cinereus*	0.31	0.18
Vombatomorphia+Phascolarctidae (ASR)	0.35 (0.24–0.45)	0.29 (0.10–0.48)
*Wakaleo pitikantensis*	0.39	N/A
*Thylacoleo carnifex*	0.35	0.34
Thylacoleonidae (ASR)	0.38 (0.34–0.42)	N/A
Vombatiformes (ASR)	0.35 (0.27–0.43)	0.30 (0.10–0.50)
*Cercartetus lepidus*	0.30	N/A
*Perameles bougainville*	0.22	0.25
*Didelphis marsupialis*	0.29	0.27

Ancestral state reconstructions (indicated by ASR) were inferred using StableTraits on the majority rule consensus with all compatible partitions of the post-burnin trees from a Bayesian analysis of our 99 character morphological matrix using the Mk*v* model; the median estimates and 95% confidence intervals (shown in brackets) from the StableTraits analyses are presented here.

^a^Estimated (see Munson^46^).

However, the humerus of *Mukupirna* (Fig. 4a) differs from those of vombatids in lacking a hypertrophied deltopectoral crest that extends laterally beyond the edge of the humeral shaft and forms a tunnel-like fossa for the origin of the brachialis muscle^17,46^.

### Phylogenetic relationships of *Mukupirna nambensis* and other vombatiforms and morphological character evolution within Vombatiformes

**Figure 5:**
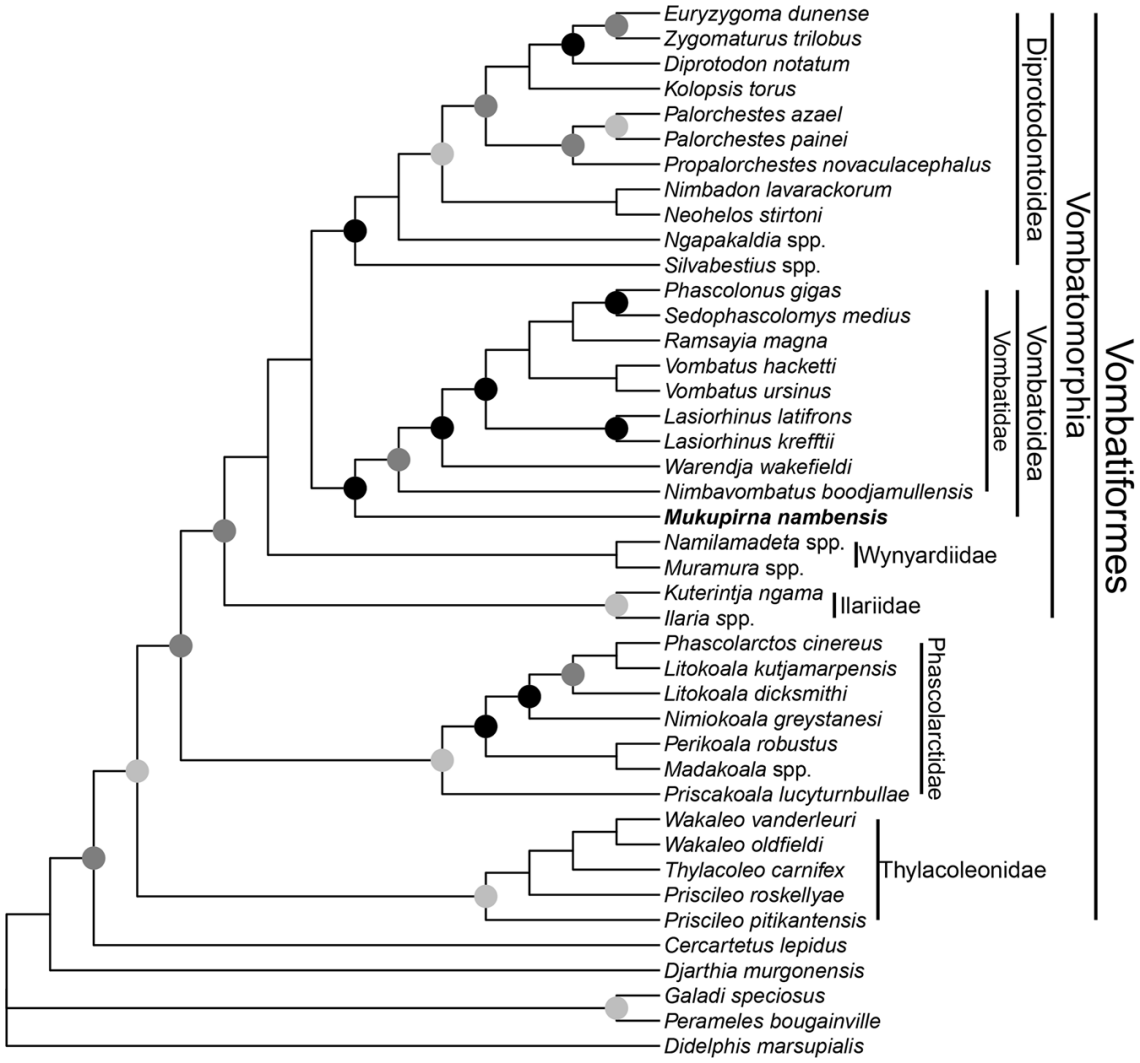
Phylogeny of Vombatiformes based on undated Bayesian analysis of a morphological dataset comprising 79 craniodental characters scored for 36 fossil and extant vombatiforms and five non-vombatiform outgroup taxa. The analysis used the Mk*v* model as implemented in MrBayes 3.2.7, with rate heterogeneity between characters modelled using an eight category lognormal distribution. The topology is a majority rule consensus of post-burn-in trees, retaining compatible partitions with Bayesian posterior probability (BPP) < 0.5. Black circles at nodes represent Bayesian posterior probability (BPP) of ≥ 0.95, dark grey circles represent BPP of 0.75-0.94 and light grey circles represent BPP of 0.5-0.74. Nodes without circles have BPP < 0.5. *Mukupirna nambensis* is shown in bold.

Phylogenetic analysis of 79 craniodental and 20 postcranial characters using undated Bayesian inference (using the Mk*v* model and an eight category lognormal distribution to accommodate rate heterogeneity between characters) places *Mukupirna* as sister to Vombatidae, with high support (Bayesian posterior probability = 0.98; Fig. 5), and hence a member of Vombatoidea as defined here (Table 1). Maximum parsimony analysis of the same matrix also recovers a *Mukupirna* + Vombatidae clade, although with relatively low support (bootstrap = 45%; see supplementary information). The Bayesian analysis and the maximum parsimony analysis identify the same four features as unambiguous synapomorphies of *Mukupirna* + Vombatidae: prominent lingual cusp on P3 absent (character 10); enamel extending down the buccal surface of P3 and onto the root present (character 11); articular eminence of glenoid fossa planar or concave, and mandibular fossa absent or indistinct (character 63); postglenoid process absent or weakly developed (character 64).

Monophyly of all currently recognised vombatiform families represented by two or more terminals is supported by BPP > 0.5, except Wynardiidae, with BPP = 0.36. Thylacoleonidae is sister to the remaining vombatiforms, rather than (as in most previous studies) a member of Vombatomorphia; however, this relationship was also found by Gillespie *et al.*^48^. Monophyly of Vombatomorphia and Phascolarctidae to the exclusion of Thylacoleonidae receives relatively strong support (BPP = 0.76). Within Vombatomorphia, Vombatoidea and Diprotodontoidea are very weakly supported as sister-taxa (BPP = 0.16), with similarly weak support (BPP = 0.26) for Wynyardiidae as sister to this clade to the exclusion of ilariids.

Ancestral state reconstructions (ASRs) of Epicondylar Index (EI) and Index of Fossorial Ability (IFA) values on the Bayesian majority rule consensus retaining all compatible partitions with BPP < 0.5 (i.e. the topology shown in Fig. 5, but with branch lengths proportional to the total estimated amount of change in the morphological character matrix) using StableTraits indicate that the high EI and IFA values shared by *Mukupirna* and vombatids are homologous; median ASR values for Vombatoidea are 0.43 for EI and 0.41 for IFA, which are very similar to the values for *Mukupirna* and *Vombatus ursinus* (Table 2).

ASRs for presence or absence of P1 and P2 on the same topology and branch lengths using a single rate Mk model in Mesquite provide strong support for the hypothesis that loss of P1 and P2 occurred within Vombatiformes after the divergence of thylacoleonids (with loss of P2 occurring independently within Thylacoleonidae), but before the split between Vombatomorphia and Phascolarctidae (Table 3). ASR of molar type using the same Mk model indicates that bunolophodonty is ancestral for Vombatiformes, with subsequent acquisition of selenodonty by the Vombatomorphia+Phascolarctidae lineage, which was retained by phascolarctids and ilariids. After the divergence of ilariids, the remaining vombatomorphians evolved selenolophodonty, which was retained by wynyardiids and vombatoids (including *Mukupirna*), with diprotodontoids subsequently evolving fully lophodont molars (Table 3). This scenario for the evolution of different molar types within Vombatiformes is attractive in its simplicity, but we acknowledge that it is partially dependent on some very weakly supported relationships, specifically the position of Wynyardiidae and Ilariidae as successive sister taxa to Diprotodontoidea + Vombatoidea.

**Table 3:** Ancestral state reconstructions (ASRs) for presence or absence of P1, presence or absence of P2, and molar type for Vombatiformes and selected vombatiform subclades (see Table 1 and Figs. 1, 2).

Clade	P1	P2	molar type
Vombatiformes	present (p = 0.98)	present (p = 0.96)	bunolophodont (p = 0.96)
Thylacoleonidae	present (p = 0.99)	present (p = 1.00)	bunolophodont (p = 0.97)
Phascolarctidae+Vombatomorphia	absent (p = 0.98)	absent (p = 0.95)	selenodont (p = 0.88)
Phascolarctidae	present (p = 1.00)	absent (p = 1.00)	selenodont (p = 1.00)
Vombatomorphia	absent (p = 1.00)	absent (p = 1.00)	selenodont (p = 0.84)
Vombatoidea	absent (p = 1.00)	absent (p = 1.00)	selenolophodont (p = 1.00)
Vombatidae	absent (p = 1.00)	absent (p = 1.00)	selenolophodont (p = 1.00)
Diprotodontoidea	absent (p = 1.00)	absent (p = 1.00)	fully lophodont (p = 1.00)

ASRs were inferred using the Mk1 (symmetrical) model in Mesquite version 3.61 on the majority rule consensus with all compatible partitions of the post-burnin trees from a Bayesian analysis of our 99 character morphological matrix under the Mk*v* model; the most likely optimisation is given for each clade, with the associated probability in brackets.

### Estimated size of *Mukupirna nambensis* and body mass evolution within Vombatiformes

Estimated body mass of *Mukupirna* using the “total skull length” regression equation of Myers^49^ is 46 kg, which seems implausibly low given the size of the postcranium. We note that the largest specimen used by Myers^49^ in calculating his regression equations was a 70 kg *Macropus* individual (species unspecified), and so use of these equations to estimate body masses of likely larger extinct taxa involves extrapolation beyond the data used to calculate them. The overall proportions of *Mukupirna* and several other extinct vombatiforms (e.g. diprotodontoids, thylacoleonids) also appear very different from the extant marsupial species used by Myers^49^ to produce the regression equations. We have therefore used the postcranial regression equations presented by Richards *et al*.^13^, which were produced using a dataset of mammalian and non-mammalian taxa with body masses that collectively span from 51 g to 6.4 tonnes. These give body mass estimates for *Mukupirna* of 143 kg based on femoral circumference only, 160 kg based on combined humeral and femoral circumference, and 171 kg based on humeral circumference only. The humerus of *Mukupirna* is very robust, and so the estimates that incorporate humeral circumference might be inflated, as Richards *et al*.^13^ also suggested for the vombatiform *Palorchestes*; nevertheless, it seems likely that *Mukupirna* exceeded 100 kg. This is compared to an average weight of 32 kg for the largest living vombatiform, the northern hairy-nosed wombat (*Lasiorhinus kreffttii*).

**Figure 6:**
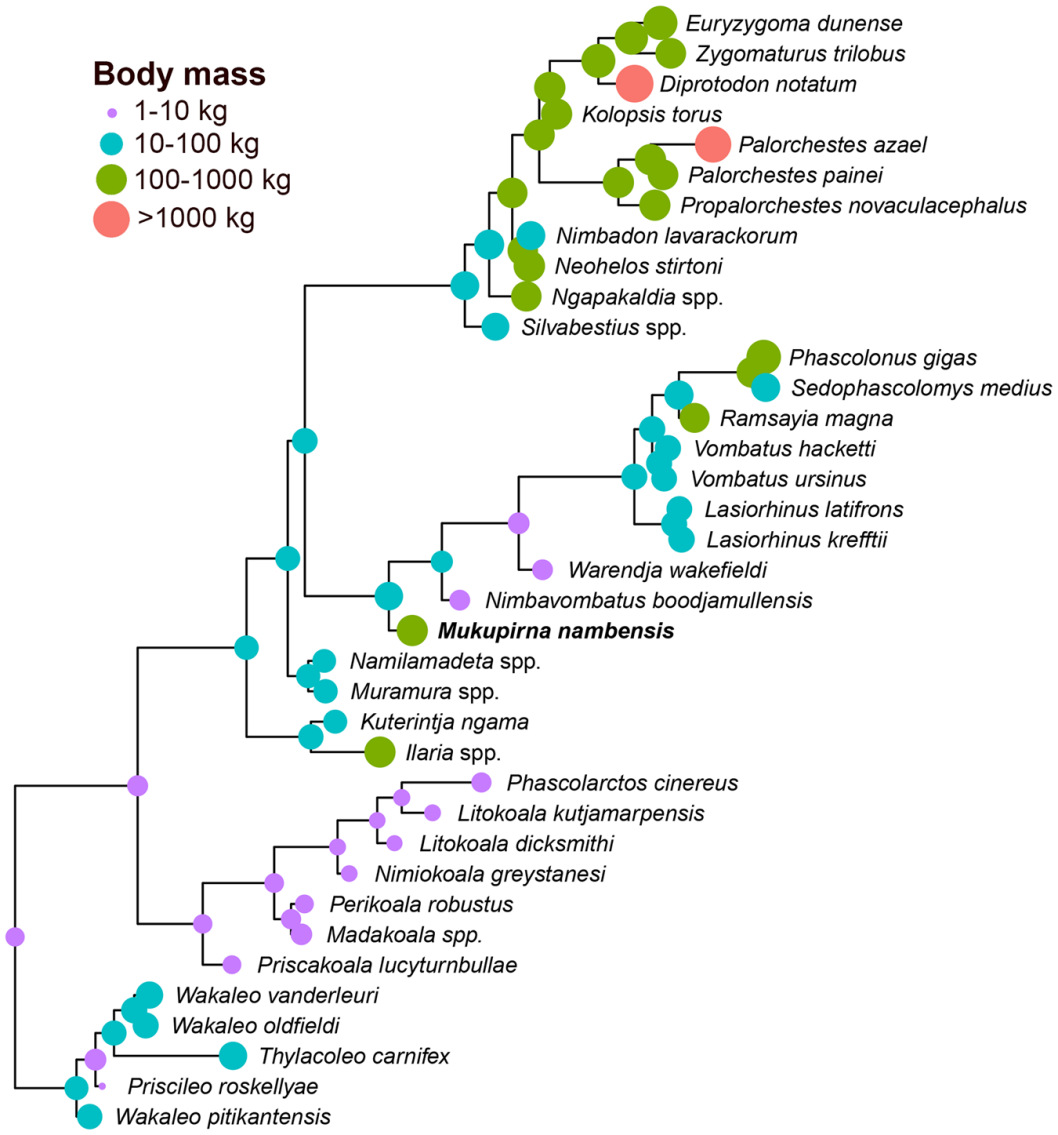
StableTraits ancestral state reconstruction (ASR) of body mass estimates for Vombatiformes. The tree used for ASR is the majority rule consensus that retains compatible partitions with BPP < 0.5 from our undated Bayesian analysis (branching topology same as in Fig. 5), with branch lengths proportional to the total estimated amount of change in our morphological characters.

Compared to estimated body masses for selected other late Oligocene vombatimorphians (see supplementary material), *Mukupirna* is much larger than the wynyardiid *Muramura williamsi* (16–20 kg), slightly larger than the diprototodontid *Ngapakaldia bonythoni* (~119 kg), but somewhat smaller than the ilariid *Ilaria illumidens* (~215 kg). Ancestral state reconstruction of body mass on the Bayesian majority rule consensus retaining compatible partitions with BPP < 0.5 (in which branch lengths are proportional to the estimated amount of change in the morphological characters used to infer the phylogeny) using StableTraits suggest a median body mass of 5.5 kg for the last common ancestor of vombatiforms, which is only slightly smaller than the modern koala *Phascolarctos cinereus* (Table 2). The thylacoleonid *Microleo attenboroughi* from the early Miocene of Riversleigh World Heritage Area has not been included here due to its comparative incompleteness; however, it has an estimated body mass of 590 g^48^, and, if it is sister to all other known thylacoleonids (as found by Gillespie *et al*.^48^), then it implies an even smaller ancestral body mass for Vombatiformes, possibly <1 kg. Our StableTraits analysis of body mass (Fig. 6) indicates independent evolution of very large (>100 kg) size at least six times within Vombatiformes. For the thylacoleonid *Thylacoleo carnifex*, we have used the mean of estimates based on postcranial measurements (= 57.2 kg^13^); however, we note other studies have found that body mass of some *T. carnifex* individuals may have exceeded 100 kg^50,51^, in which case it would represent a seventh independent evolution of >100 kg body mass within Vombatiformes.

## Discussion

Our phylogenetic results imply that postcranial digging adaptations, such as large EI and IFA, evolved in Vombatoidea in an ancestor that still retained a somewhat wynyardiid-like, selenolophodont molar dentition (as seen in *Mukupirna* and also the plesiomorphic vombatids *Nimbavombatus* and *Rhizophascolonus*), rather than the specialised, hypselodont molars characteristic of later vombatids. However, *Mukupirna* lacks a laterally extensive, flange-like deltopectoral crest, which is present in all vombatids known from associated postcranial material^17^. This modified deltopectoral crest creates an enclosed, tunnel-like fossa for the origin of the brachialis muscle, and is likely a specialised fossorial adaptation^17^. Absence of this feature in *Mukupirna*, together with its large size (>100 kg) means that *Mukupirna* may not have been capable of the true burrowing behaviour of modern wombats^17,46,52^. Instead, it may have used scratch-digging to access subterranean food items, such as roots and tubers, as has also been proposed for *Ilaria*^46^ and *Rhizophascolonus*^36^.

It has been suggested, based on the ecology and relatively close relationship of modern wombats and koalas, that vombatiforms (and also other diprotodontians) have been characterised by “long-term maintenance of ecological niche differentiation”^53^. However, the evidence from the fossil record of Vombatiformes clearly demonstrates that this is an artefact of the relictual nature of the three extant representatives: known fossil vombatiforms range in size from small (<5 kg) Oligo-Miocene phascolarctids^32^ and thylacoleonids^48,54^, to the rhino-sized (> 2 tonne) *Diprotodon* from the Pleistocene, and collectively span a diverse range of morphologies and ecologies, including several that lack obvious modern analogues (at least in the Australian mammal fauna). The >100 kg *Mukupirna*, together with similarly sized contemporaneous taxa such as the ilariid *Ilaria* and the diprotodontid *Ngapakaldia*, demonstrates that multiple vombatiform lineages had already evolved very large (>100 kg) body size by the late Oligocene, and possibly considerably earlier. In total, our ancestral state reconstructions indicate at least six independent origins of body masses>100 kg within Vombatiformes, from an ancestor that is estimated to have been between 1 and 5.5 kg, the upper bound being slightly smaller than the mean mass of the living koala, *Phascolarctos cinereus*. In this respect, vombatiforms resemble another mammalian clade with relictual modern diversity but for which far more diverse fossil representatives are known, namely sloths^55^.

## Material and Methods

### Phylogenetic analysis

To resolve the phylogenetic relationships of *Mukupirna nambensis*, we used a novel morphological dataset of 79 craniodental and 20 postcranial characters, focused on vombatiforms and largely based on the matrix of Brewer *et al*.^20^. All multistate characters representing putative morphoclines were specified as ordered.

Undated Bayesian analysis of the morphological dataset was carried out using MrBayes 3.2.7^56^, using the Lewis^57^ Mk model with the assumption that only variable characters were scored (i.e. the Mk*v* model), and with rate heterogeneity between characters modelled using an eight category lognormal distribution^58^. The Bayesian analysis were run for 10 × 10^6^ generations, using four independent runs of four chains (one cold and three heated chains, with the temperature of the heated chains set to the default value of 0.1), and sampling trees every 5000 generations. Tracer 1.7^59^ was then used to identify an appropriate burn-in period for the trees saved for each run. The post-burn-in trees were then summarised using MrBayes in the form of a majority rule consensus that retains compatible partitions with BPP < 0.5, using the “contype = allcompat” comand. The matrix was analysed using maximum parsimony, in TNT version 1.5, with the tree search comprising an initial “new technology” search with sectorial search, ratchet, drift and tree fusing that was run until the same minimum tree length was found 1000 times, followed by a “traditional” search within the trees save from the first stage, using the tree bisection resection (TBR) swapping algorithm. Multiple most parsimonious trees were combined using strict consensus. Support values were calculated using 2000 standard bootstrap replicates, implemented using a “traditional” search, with results output as absolute frequencies.

### Estimation of body mass

To estimate body mass of *Mukupirna nambensis* and for other fossil vombatiforms that lack published estimates but for which appropriate postcranial material was available, we used the regression equations presented by Richards *et al*.^13^, which are based on humeral circumference, femoral circumference, and combined humeral and femoral circumference. Where possible, we have preferred estimates based on these measurements rather than those based on craniodental measurements (e.g., using the regression equations of Myers^49^), as it seems more likely that circumference of the limb bones will more accurately reflect body mass than will dimensions of the skull and teeth^60^. Where at least one humerus and one femur was available for a particular taxon, we used all three of the Richards *et al*.^13^ equations, and calculated a mean value.

For taxa for which postcranial material was not available, we used the craniodental regressions of Myers^49^, using his “diprotodontians” dataset. For each taxon, we used up to four of the highest ranking (as measured by total rank) equations that could be calculated based on available material, incorporating the relevant smearing estimates, and then used these estimates to calculate a mean value.

### Ancestral state reconstructions of continuous traits

For ancestral state reconstruction of continuous variables – namely Epicondylar Index (EI), Index of Fossorial Ability (IFA) and body mass -, we used the “stable” model implemented StableTraits 1.5^61^, which allows occasional “jumps” in trait values, and which is likely to outperform a standard Brownian motion model^61,62^. StableTraits requires a fully resolved phylogeny with branch lengths, and so we used a majority rule consensus that retains compatible partitions with BPP < 0.5 that resulted from our undated Bayesian analysis, in which branch length is proportional to the total estimated amount of change in our morphological characters. StableTraits cannot be used with zero-length branches, and so we increased the length of all such branches by a trivial amount (10^−6^). For each StableTraits analysis, taxa that lacked data were deleted. Values for EI and IFA (which are ratios) were used “as is”, but body mass was log10-transformed prior to analysis. Each StableTraits analysis was run using default settings: two independent runs of 1 × 10^6^ generations, in 40 stages of 2.5 × 10^4^ generations. The first 5 × 10^5^ generations (i.e. 50%) were discarded as burn-in; the proportional scale reduction factor PRSF,^63^ was <1.1 (indicating convergence) before this point.

### Ancestral state reconstructions of discrete dental traits

We also used the majority rule consensus that retains compatible partitions with BPP < 0.5 from our undated Bayesian analysis, with branch lengths proportional to the total estimated amount of change in our morphological characters, to implement ancestral state reconstruction of the following discrete dental traits: presence or absence of P1, presence or absence of P2, and molar type. The first two traits were taken directly from the morphological dataset we used in our phylogenetic analysis (Characters 4-5), whilst the third reflects overall molar morphology, with the following states: tribosphenic, bunodont (i.e., distinct crests absent), bunolophodont (i.e., weakly developed lophs present), fully lophodont (i.e., tall, well-developed lophs present), selenodont, and selenolophodont (i.e., both selenodont and lophodont features present). Ancestral state reconstruction was done in Mesquite version 3.61^64^, using the Mk1 (symmetrical) model.

### Nomenclatural acts

This published work and the nomenclatural acts it contains have been registered in ZooBank, the proposed online registration system for the International Code of Zoological Nomenclature (ICZN). The ZooBank LSIDs (Life Science Identifiers) can be resolved and the associated information viewed through any standard web browser by appending the LSID to the prefix “http://zoobank.org/“. The LSIDs for this publication are: urn:lsid:zoobank.org:act:7303DC9B-AD03-4BB1-985D- 35F2BBE52EBE; urn:lsid:zoobank.org:act:6281C654-D711-459D-93E8-955449B645FD; urn:lsid:zoobank.org:act:CC66EA70-9914-4826-A491-09DBFAF4C00A.

## Supplementary information

Supporting information.
