# Supplementary material for: A new family of diprotodontian marsupials from the latest Oligocene of Australia and the evolution of wombats, koalas, and their relatives (Vombatiformes)
Source: Sci Rep. 2020 Jun 25;10:9741. doi: 10.1038/s41598-020-66425-8 (PMC7316786; doi:10.1038/s41598-020-66425-8)
Supplement: Supplementary file 1 — Supporting information. [file 41598_2020_66425_MOESM1_ESM.docx]

SUPPLEMENTARY INFORMATION FOR

**A new family of diprotodontian marsupials from the latest Oligocene of Australia and the evolution of wombats, koalas, and their relatives (Vombatiformes)**

Robin M. D. Beck^1,2*^, Julien Louys^3^, Philippa Brewer^4^, Michael Archer^2^, Karen H. Black^2^, Richard H. Tedford^5^ (deceased)

^1^Ecosystems and Environment Research Centre, School of Science, Engineering and Environment, University of Salford, Manchester, UK

^2^PANGEA Research Centre, School of Biological, Earth and Environmental Sciences, University of New South Wales, Sydney, New South Wales, Australia

^3^Australian Research Centre for Human Evolution, Environmental Futures Research Institute, Griffith University, Queensland, Australia

^4^Department of Earth Sciences, Natural History Museum, London, United Kingdom

^5^Division of Paleontology, American Museum of Natural History, New York, USA

Correspondence and requests for materials should be addressed to R.M.D.B (email: r.m.d.beck@salford.ac.uk)

**This pdf includes:**

**Supplementary figures**

**Supplementary tables**

**Comparative material**

**Full description**

**Relevance of *Marada arcanum***

**List of morphological characters**

**Morphological matrix in NEXUS format**

**Justification for body mass estimates**

**References**

**Figure S1.** Rostrum of holotype and only known specimen of *Mukupirna nambensis* gen. et. sp. nov. (AMNH FM 102646) in ventromedial (a) and anteroventral (b) views. Abbreviations: C1a, upper canine alveolus; I1a, first upper incisor alveolus; I2a, second upper incisor alveolus; I1a, third upper incisor alveolus; P3, third upper premolar. Scale bar = 1 cm.

**
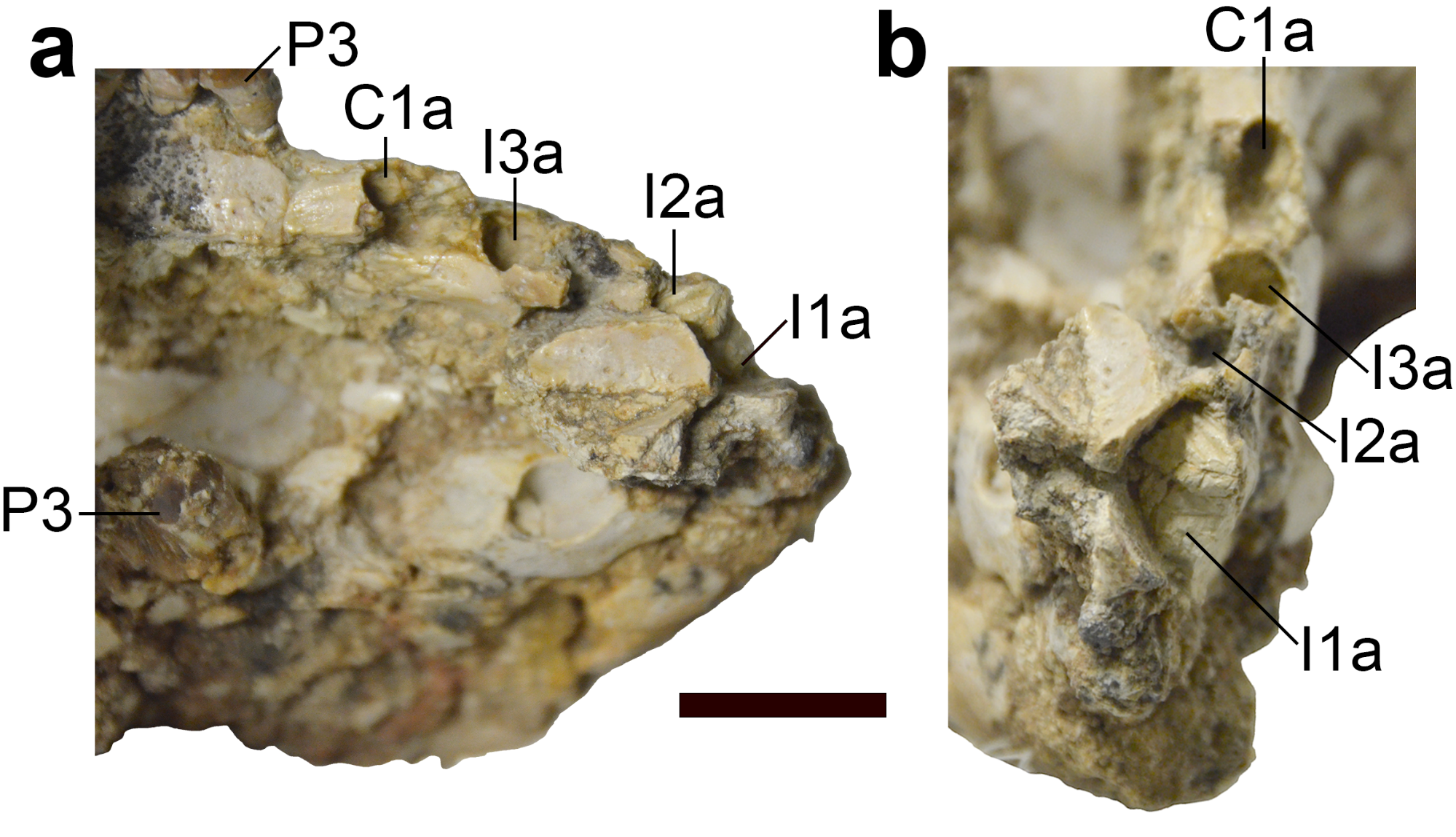
**

**Figure S2.** Postcanine dentition of holotype and only known specimen of *Mukupirna nambensis* gen. et. sp. nov. (AMNH FM 102646): right P3-M4 in occlusolingual (a), labial (b) and lingual (c) views; left P3-M4 in occlusal view (d). Scale bar = 1 cm.

**
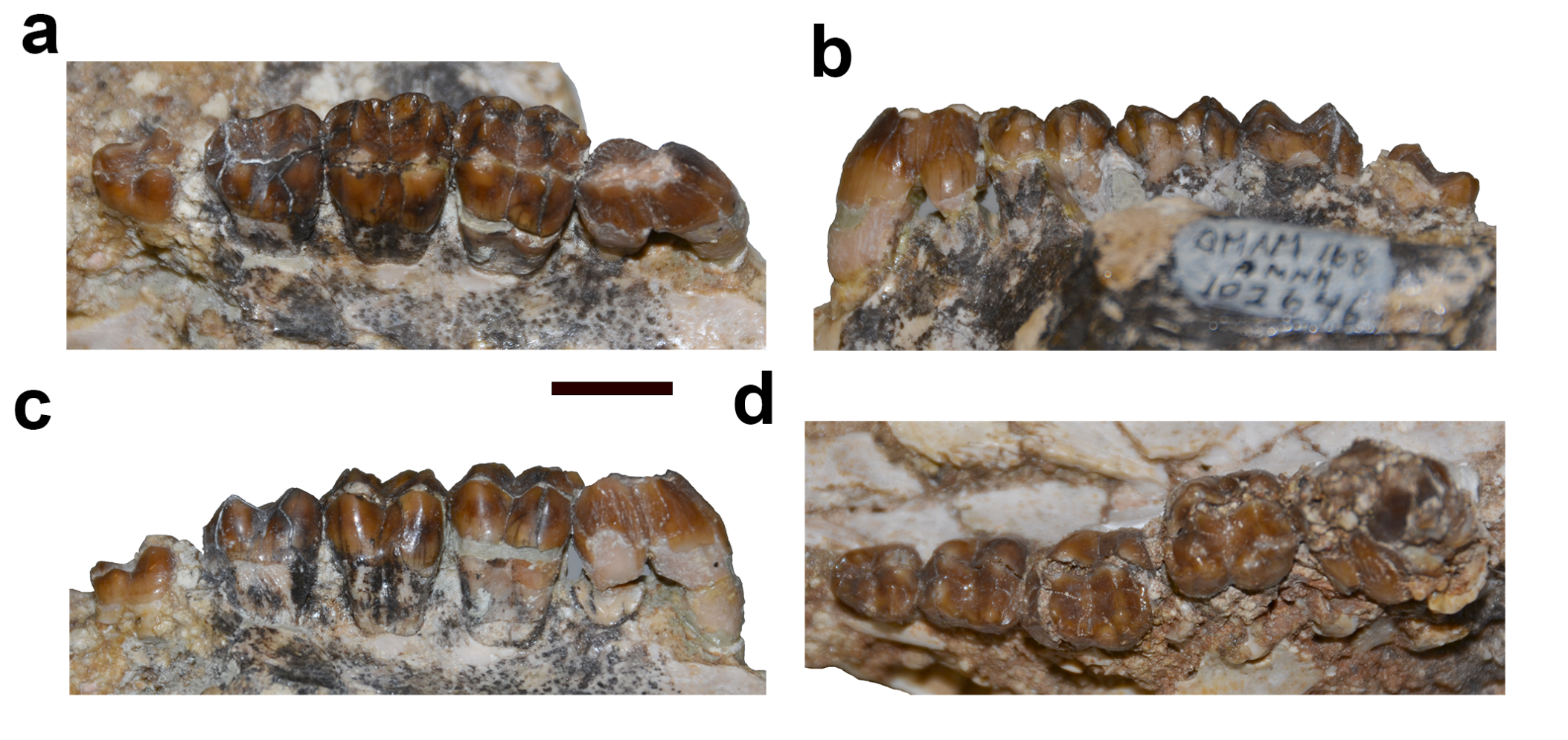
**

**Figure S3.** Scapulae of holotype and only known specimen of *Mukupirna nambensis* gen. et. sp. nov. (AMNH FM 102646): right scapula in lateral view (a); left scapula in lateral view (b); right scapula in distal view (c); left scapula in medial view (d). Abbreviations: ac, acromion; cop, coracoid process; gc, glenoid cavity; inf, infraspinatus fossa; sgt, supraglenoid tubercle; ssf supraspinatus fossa; ssp, scapular spine. Scale bar = 5 cm.

**
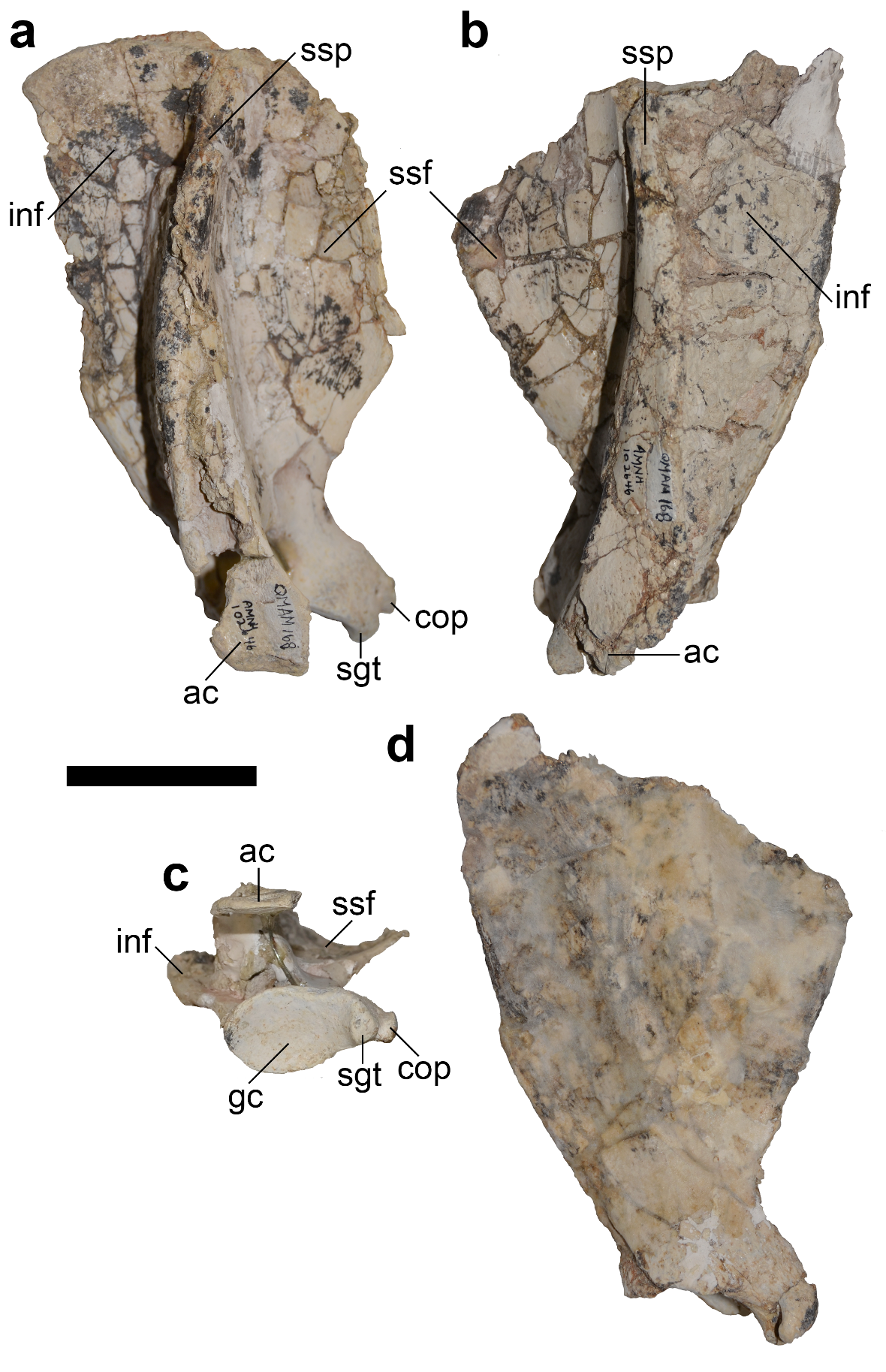
**

**Figure S4.** Left ulna of holotype and only known specimen of *Mukupirna nambensis* gen. et. sp. nov. (AMNH FM 102646): anterior (a), medial (b) and lateral (c) views of entire element; anteromedial (d) and anterolateral (e) views of proximal end. Abbreviations: anp, anconeal process; cop, coronoid process; fls, flexor sulcus; ol, olecranon; rn, radial notch; trn, trochlear notch. Scale bar in a-c = 5 cm; scale bar in d-e = 2 cm.

**
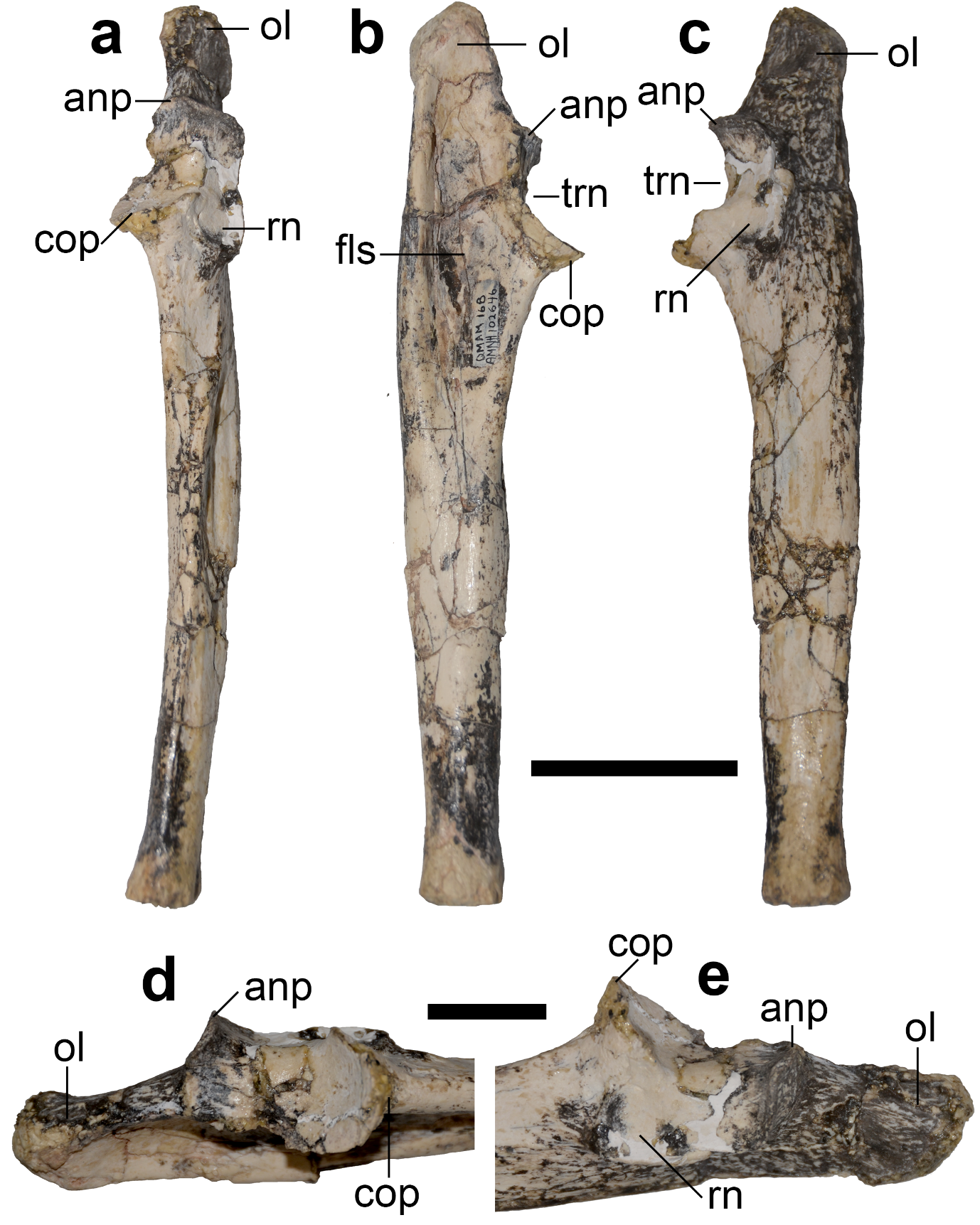
**

**Figure S5.** Left tibia of holotype and only known specimen of *Mukupirna nambensis* gen. et. sp. nov. (AMNH FM 102646) in anterior (a), lateral (b), medial (c) and proximal (d) views. Abbreviations: ie, intercondylar eminence; lco, lateral condyle; mco, medial condyle; tcr, tibial crest. Scale bar = 5 cm.


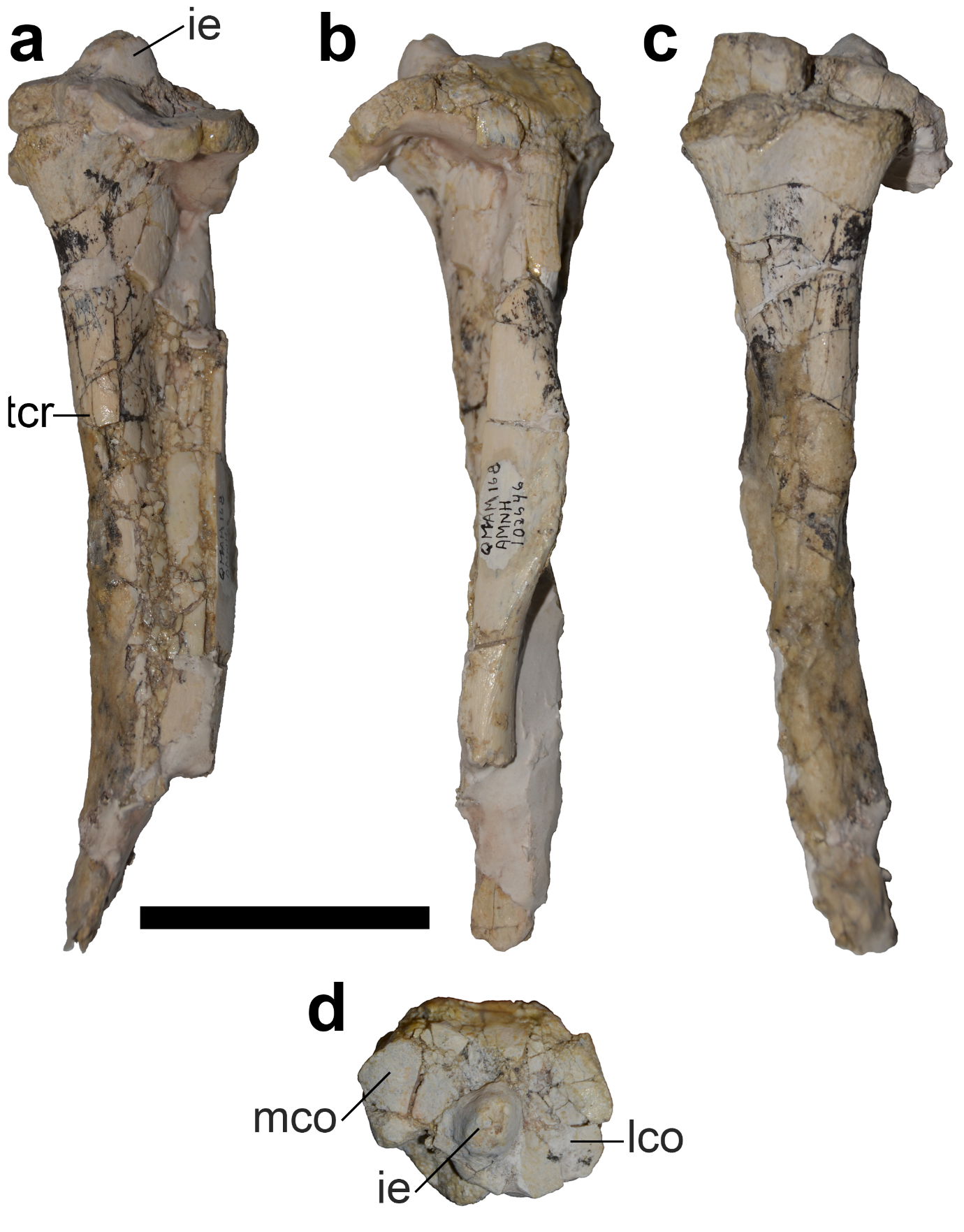


**Figure S6.** Majority rule consensus of post-burn-in trees, retaining compatible partitions with Bayesian posterior probability (BPP) <0.5, following Bayesian analysis of our 99 character morphological character matrix using the Mk*v* model, as implemented by MrBayes 3.2.7 (see Figures 5-6 of the main text). Numbers at nodes represent Bayesian posterior probabilities.

**
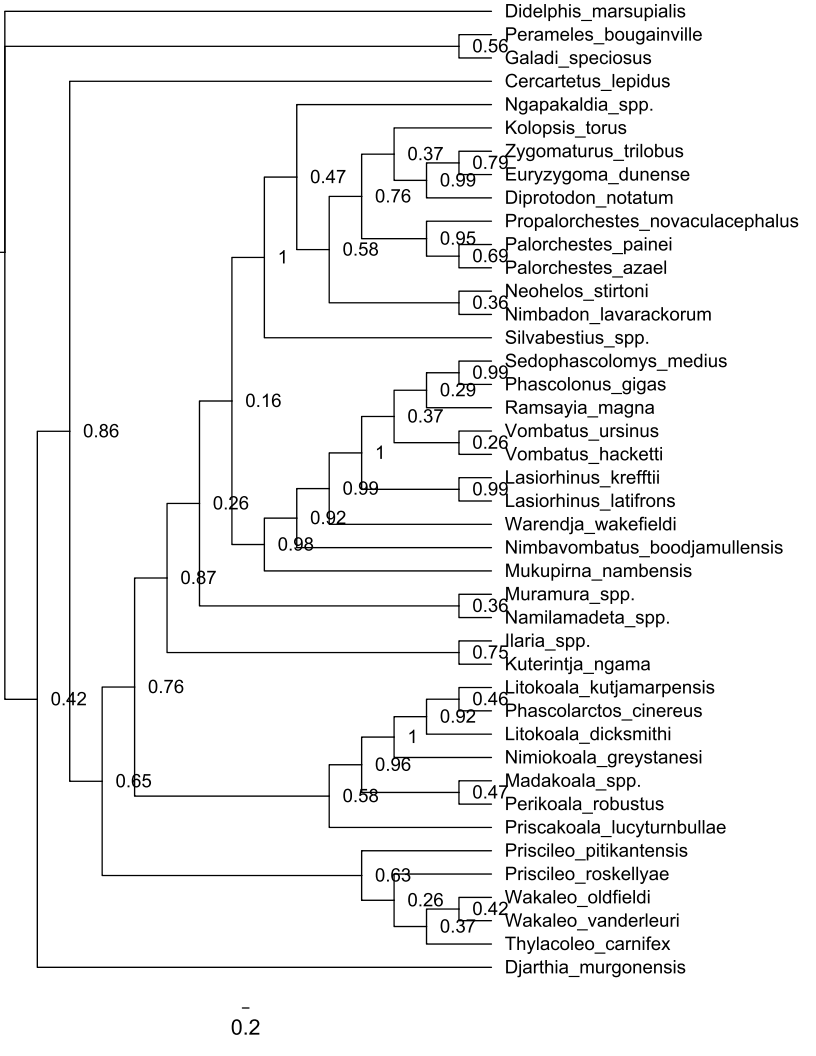
**

**Figure S7.** Strict consensus of 504 most parsimonious trees (length = 244 steps) that result from maximum parsimony analysis using TNT of our 99 character morphological character matrix. Numbers at nodes represent bootstrap support values (2000 standard replicates using traditional search, with results output as absolute frequencies).

**
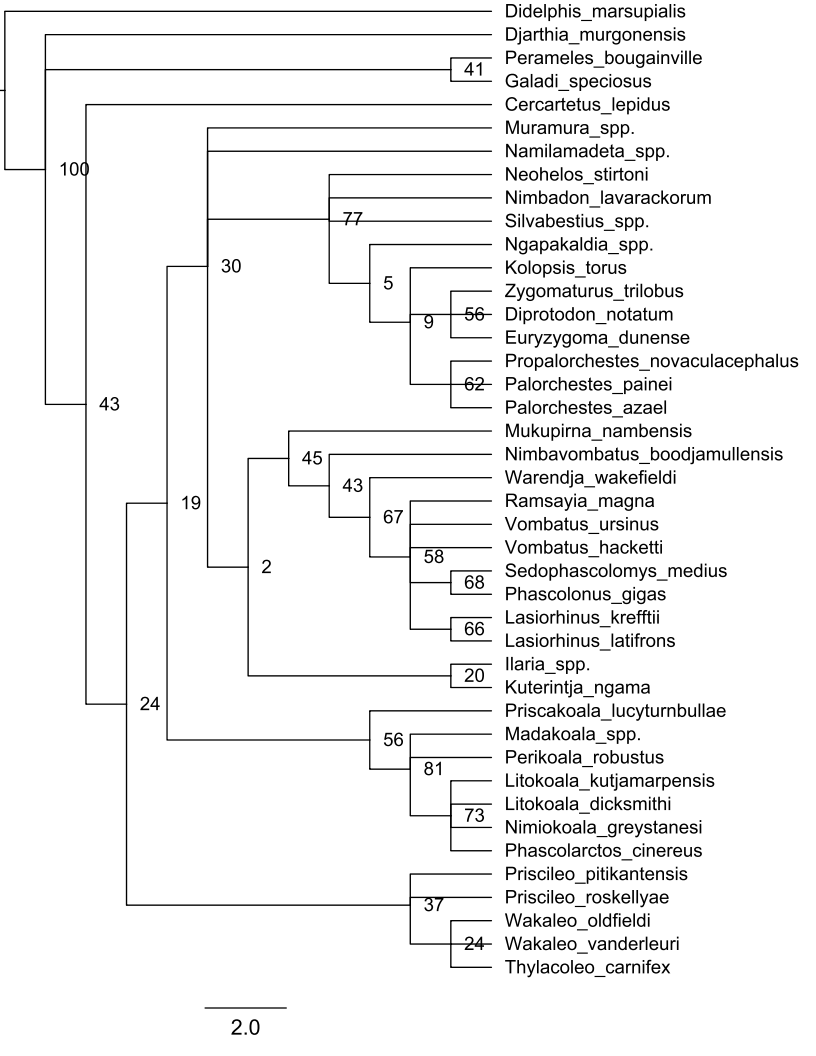
**

**Supplementary tables**

**Table S1.** Measurements of postcanine dimensions in holotype and only known specimen of *Mukupirna nambensis* (AMNH FM 102646).

|  | **length** | | **anterior width** | | **posterior width** | |
| --- | --- | --- | --- | --- | --- | --- |
| **tooth** | **left** | **right** | **left** | **right** | **left** | **right** |
| P3 |  | 12.4 |  | 7.3 |  | 8.9 |
| M1 |  |  | 10.6 | 9.9 | 10.4 | 10.2 |
| M2 | 12.1 | 11.4 | 9.8 (approx.) | 9.6 | 10.2 (approx.) | 9.1 |
| M3 | 9.6 | 9.9 | 9.3 | 9.8 | 8.3 | 8.4 |
| M4 | 8.2 | 8.9 | 7.2 | 5.8 (approx.) | 5.4 | 5.6 |

**Table S2.** Estimates of Shoulder Moment Index (deltopectoral crest length/humeral length) for *Mukupirna nambensis* and a range of other vombatiforms.

| **Taxon** | **Shoulder Moment Index** |
| --- | --- |
| *Phascolonus gigas* | 0.65 |
| *Palorchestes azael* | 0.62 |
| ***Mukupirna nambensis*** | **0.60** |
| *Vombatus ursinus* | 0.58 |
| *Zygomaturus trilobus* | 0.56 |
| *Lasiorhinus latifrons* | 0.56 |
| *Muramura williamsi* | 0.55 |
| *Thylacoleo carnifex* | 0.53 |
| *Diprotodon opatum* | 0.52 |
| *Phascolarctos cinereus* | 0.48 |
| *Ngapakaldia tedfordi* | 0.40 |

**Table S3.** Body mass estimates of vombatiform and non-vombatiform marsupials. See “Justification for body mass estimates” below.

| **Taxon** | **body mass (g)** | **log10(body mass)** |
| --- | --- | --- |
| *Didelphis marsupialis* | 1134.75 | 3.0549 |
| *Djarthia murgonensis* | 42.3 | 1.62634 |
| *Perameles bougainville* | 230.8 | 2.363236 |
| *Galadi speciosus* | 917.5 | 2.962606 |
| *Cercartetus lepidus* | 8.03 | 0.904716 |
| *Ngapakaldia* spp. | 119200 | 5.076276 |
| *Kolopsis torus* | 156000 | 5.193125 |
| *Neohelos stirtoni* | 173300 | 5.238799 |
| *Nimbadon lavarackorum* | 70000 | 4.845098 |
| *Litokoala kutjamarpensis* | 3700 | 3.568202 |
| *Litokoala dicksmithi* | 3300 | 3.518514 |
| *Madakoala* spp. | 9000 | 3.954243 |
| *Nimiokoala greystanesi* | 3600 | 3.556303 |
| *Perikoala robustus* | 5100 | 3.70757 |
| *Phascolarctos cinereus* | 6528.74 | 3.814829 |
| *Wakaleo pitikantensis* | 23300 | 4.367356 |
| *Priscileo roskellyae* | 1813 | 3.258398 |
| *Wakaleo oldfieldi* | 28400 | 4.453318 |
| *Wakaleo_vanderleuri* | 41400 | 4.617 |
| *Sedophascolomys medius* | 70000 | 4.845098 |
| *Phascolonus gigas* | 599250 | 5.777608 |
| *Ramsayia magna* | 100000 | 5 |
| *Lasiorhinus krefftii* | 31849.99 | 4.503109 |
| *Lasiorhinus_latifrons* | 26163.8 | 4.417701 |
| ***Mukupirna nambensis*** | **157999.6** | **5.198656** |
| *Vombatus ursinus* | 26000 | 4.414973 |
| *Vombatus hacketti* | 30000 | 4.477121 |
| *Warendja wakefieldi* | 7500 | 3.875061 |
| *Muramura spp.* | 17749.36 | 4.249183 |
| *Priscakoala lucyturnbullae* | 5100 | 3.70757 |
| *Zygomaturus trilobus* | 128400 | 5.108565 |
| *Diprotodon notatum* | 2428400 | 6.38532 |
| *Euryzygoma dunense* | 500000 | 5.69897 |
| *Propalorchestes novaculacephalus* | 155400 | 5.191451 |
| *Palorchestes_painei* | 128331.83 | 5.108334 |
| *Palorchestes azael* | 1254700 | 6.09854 |
| *Silvabestius spp.* | 49627.89 | 4.695726 |
| *Namilamadeta spp.* | 14961.65 | 4.174979 |
| *Ilaria spp.* | 154669.02 | 5.189403 |
| *Kuterintja_ngama* | 16074.57 | 4.206139 |
| *Thylacoleo carnifex* | 57250 | 4.757775 |
| *Nimbavombatus boodjamullensis* | 7500 | 3.875061 |

**Comparative material**

We used a wide range of specimens of extant and fossil vombatiforms, plus the non-vombatiform diprotodontian *Trichosurus vulpecula*, for comparative purposes, as listed below.

Institutional Abbreviations: AM, Australian Museum; AR, Archer Collection (University of New South Wales); CPC, CPC, Commonwealth Palaeontological Collection; FU, Flinders University palaeontology collection; MAGNT, Museum and Art Gallery of the Northern Territory; NTM, Northern Territory Museum; QM, Queensland Museum; QVM, Queen Victoria Museum; SAM, South Australian Museum; SGM, Spencer and Gillen Museum (Northern Territory Museum); UCMP, University of California Museum of Paleontology; UNSWZ, University of New South Wales zoological collection

*Alkwertatherium webbi*: NTM P883, NTM P888 (craniodental)

*Diprotodon optatum*: QM F535, QM F1409, QM F1519, QM F6633 (cast), QM F10311, QM F11636, QM F25304 (craniodental); AM F99643, AM F119223, FU 2088, FU 2116, FU 2135, FU 2136, FU 2196, FU 2202, FU 2213, FU 2114, FU 2219, FU 2224, SAM P5037, SAM P5038, SAM P5039, SAM P5041, SAM P5043, SAM P5120, SAM P5121, SAM P5122-5183, SAM P5144, SAM P5149, SAM P6780, SAM P10550-10569, SAM P11523, SAM P12308, SAM P12309, SAM P20831, SAM P20832, SAM P20833, SAM P20834, SAM P20835, SAM P20868, SAM P20875, SAM P20876, SAM P20877, SAM P20880, SAM P20882, SAM P20894, SAM P20895, SAM P25085, SAM P25266, SAM P25285, SAM P25314, SAM P25315, SAM P25316, SAM P25317, SAM P25328, SAM P29257, SAM P60789, SAM P60836 (postcranial)

*Euowenia grata*: QM F519, QM F3824, QM F3842, QM F12480, QM F12481 (craniodental); AM F4884, AM F4885, FU 2399, FU 2671 (postcranial)

*Euryzygoma dunense*: QM F376, QM F2114, QM F3328, QM F3335, QM F3362, QM F3367, QM F3370, QM F6141, QM F12482 (craniodental)

*Hulitherium tomasettii*: AM F73516 (cast), AM F73517 (cast), AM F81854 (cast) (craniodental)

*Kolopsis rotundus*: AM F38574, AM F41443, AM F41444, AM F44470, AM F44471 (craniodental)

*Kolopsis torus:* CPC 6747, SGM 889 (craniodental); MAGNT Jul-92, MAGNT MP87, MAGNT MP274, MAGNT MP581, MAGNT MP606, MAGNT MP628, MAGNT MP660, MAGNT MP677, MAGNT MP936A, MAGNT MP936B, MAGNT MP936D, MAGNT MP1039, MAGNT MP1081, MAGNT MP1096, MAGNT MP1108, MAGNT MP1153, MAGNT MP1159, MAGNT MP1160, MAGNT MP1162, MAGNT MP1164, MAGNT NP5, MAGNT P9290, MAGNT SP257, MAGNT SQ304, MAGNT SQ417, MAGNT SQ2006, NTMGA MP606 (postcranial)

*Kolopsis yperus*: NTM P92136, NTM P92115, NTM P92117 (craniodental)

*Kolopsoides cultridens*: UCMP 67601 (cast) (craniodental)

*Kuterintja ngama*: QM F20810, QM F23203, QM F30057, QM F30058, QM F31299,

QM F40324

*Lasiorhinus latifrons*: SAM M245, SAM M299, SAM M395, SAM M1926, SAM M2063, SAM M2107, SAM M2108, SAM M2109, SAM M2110, SAM M2111, SAM M2165, SAM M2719, SAM M2934, SAM M5244, SAM M8663, SAM M14038, SAM M21407, SAM M21747, SAM M22814, SAM M23218, SAM M23219 (postcranial)

*Litokoala kutjamarpensis*: AR 19361, QM F13079, QM F20809, QM F30500, QM F30501, QM F51382 (craniodental)

*Maokopia ronaldi*: AM F83407, AM F113159, AM F113160, AM F113166, AM F113173, AM F113179, AM F119135, AM F119136, AM F119138 (craniodental)

*Meniscolophus mawsoni*: SAM P13647 (cast) (craniodental)

*Ngapakaldia bonythoni*: SAM P13863/UCMP 57258, SAM P23052 (postcranial)

*Neohelos stirtoni*: NTM P8551-13, NTM P8695-74, NTM P8697-1, NTM P87018-1 (craniodental); NMV 2328, NMV 2329; NMV 2335, NMV 2337 (postcranial)

*Neohelos tirarensis*: AM F87625, AM F87626, SAM P13848 (craniodental)

*Ngapakaldia bonythoni*: SAM P13863 (craniodental)

*Ngapakaldia tedfordi*: NMV P157585 (cast), SAM P13851, UCMP 57254 (cast) (craniodental)

*Nimbadon lavarackorum*: NMV P186506, QM F23141-23155, QM F23160, SGM 892 (craniodental); AR 17902, AR 17904, AR 18173, QM F41097, QM F41102, QM F41104, QM F41108, QM F41110, QM F41128, QM F41201, QM F41202, QM F41227, QM F41229, QM F41288, QM F50411, QM F50436, QM F50438, QM F50447, QM F50482, QM F50111, QM F50505, QM F50522, QM F50547, QM F50608, QM F50661 (postcranial)

*Nimiokoala greystanesi*: QM F23027, QM F29624, QMF30232, QM F30482, QM F30483, QM F30487, QMF30493 (craniodental)

*Palorchestes azael*: AM F452, QM F774 (cast), QM F30882 (craniodental)

*Palorchestes painei*: CPC 6752, QM F9179, UCMP 66596 (cast) (craniodental)

*Palorchestes parvus*: QM F783 (cast), QM F784 (cast), QM F789 (cast), QM F2968, QM F12476, QM F20859 (craniodental)

*Phascolonus gigas*: SAM P5021, SAM P5022, SAM P5024, SAM P5026, SAM P5027, SAM P5029, SAM P5030, SAM P5031, SAM P5039, SAM P5040, SAM P5041, SAM P5042, SAM P5043, SAM P5044, SAM P33459, SAM P33462, SAM P33463, SAM P33467, SAM P33468, SAM P P33469, SAM P33470, SAM P P33471, SAM P36279 (postcranial)

*Pitikantia dailyi*: SAM P13862 (craniodental)

*Plaisiodon centralis*: CPC 6748, SGM 871, SGM 881 (craniodental); MAGNT MP162, MAGNT MP551, MAGNT MP936D, MAGNT MP1047, MAGNT MP1098, MAGNT MP1101, MAGNT MP1153, MAGNT MP1160, MAGNT MP1162, MAGNT MP1161, MAGNT MP1164, MAGNT MP2005, MAGNT SP366 (postcranial)

*Priscileo roskellyae*: QM F20191, QM F23453, QM F29623 (craniodental)

*Propalorchestes novaculacephalus*: NMV P187282, NTM P8552-10, NTM P862-27 (craniodental)

*Pyramios alcootensis*: CPC 6749, SGM 872, SGM 891 (craniodental)

*Raemeotherium yatkolai*: SAM P19764 (cast) (craniodental)

*Thylacoleo carnifex*: 5 semi-complete, articulated individuals from Kamatsu Cave, Naracoorte (no numbers) (postcranial)

*Trichosurus vulpecula*: AM F3094, AM F12686, AR 6060, UNSWZ 48, UNSWZ 49

*Vombatus ursinus*: AR 1625, AR 1629, AR 5430, AR 7183, UNSWZ 3 (craniodental)

*Wakaleo oldfieldi*: QM F20895, SAM P17925 (craniodental)

*Warendja wakefieldi*: SAM P48980

*Zygomaturus trilobus*: AR 229, QM F6560 (cast), QMF12489 (craniodental); specimen from Mowbray Swamp housed in the Hobart Museum (no number), QVM GFU86, QVM GFU155, QVM1992 GFV4, QVM1992 GFV5, QVM1992 GFV60, QVM1992 GFV63, SAM P20812, SAM P25626, SAM P32673, SAM P75627 (postcranial)

**Full description**

*Dentition*

The description of the dentition is based largely on the right side of AMNH FM 102646, as it is somewhat better preserved overall than is the left side; differences between the right and left sides are noted where relevant.

The alveoli for the right I1-I3 are moderately well preserved, but some crushing of the I1 and I2 alveoli prevents accurate measurement of their size. The I2 alveolus is better preserved on the left side; it is 13 mm long anteroposteriorly and 8.8 mm wide buccolingually. The right I3 alveolus is somewhat better preserved than those for I1 and I2, but it is crushed anteriorly; its maximum buccolingual width is 8.6 mm. The anterior part of the C1 alveolus is damaged, but it is 6.3 mm wide buccolingually. The diastema separating C1 and P3 is relatively short (~10 mm).

The P3 is subtriangular in occlusal outline, being slightly wider posteriorly than anteriorly. Its occlusal surface is worn or damaged, but it is likely that two major cusps were present. The tooth is somewhat bulbous but also semi-trenchant (bladelike), and the cusps appear reclined posteriorly in lateral view, with the anterior cusp being particularly reclined posteriorly. Anterior and posterior cusps were probably connected by a crest, although the robustness of that crest is difficult to determine on the basis of the preserved tooth. The P3 lacks any posterolingual cusp (= “hypocone” sensu ^1,2^, unlike all wynyardiids. However the lingual cingulum is chipped, and it is possible that this species had an extremely reduced posterolingual cusp. There are distinct, fine ridges on the buccal surface of the P3; somewhat similar ridges can be observed on the P3 of phascolarctids and wynyardiids, and they are also present on the cheekteeth of some eutherian “condylarths” (e.g. *Periptychus*)^3,4^, where they have been suggested to be an adaptation to strengthen the enamel^5^.

The M1 is square in occlusal outline and bunodont, with the arrangement of cusps broadly resembling that seen in *Namilamadeta* species^2,6^. The protocone is the largest cusp in terms of occlusal area. A short, poorly developed preprotocrista connects the protocone to the anterolabial cingulum. A well-developed transverse crest connects the protocone and paracone. The paracone is cone-shaped, and it is linked to an anteriorly positioned stylar cusp (stylar cusp A, or the parastyle) by a short but well-developed, anteriorly-directed crest that is presumably the homologue of the preparacrista. Further posteriorly, there is a well-developed, conical stylar cusp in the C position (i.e. posterobuccal to the paracone), that is connected to the paracone by a short but fairly well developed postparacrista. The metacone is prominent, and also conical in shape. The anterobuccally directed premetacrista connects the metacone to a strongly developed stylar cusp D. Posterior to stylar cusp D, there is reduced stylar cusp E, which tapers posteriorly to merge with the well-developed posterior cingulum. This cingulum forms the posterior margin of the tooth; lingually, it curves ventrally to contact the metaconular hypocone at its apex. The metaconular hypocone and metacone are linked by a strongly developed transverse crest.

The M2 appears somewhat more selenodont than M1, as the parastyle is positioned more directly buccal (rather than anterobuccal) due the paracone. The paracone connects to the parastyle via a short, poorly-developed buccally-directed preparacrista. The parastyle is separated from a stylar cusp in the C position by a distinct but very narrow valley on the buccal face of the tooth. Stylar cusp C is a well-developed, rounded cusp positioned posterobuccal to the paracone, in a similar position to that seen on M1. The paracone connects to stylar cusp C via a strongly developed, L-shaped postparacrista. Similarly to the condition on M1, a strongly developed transverse crest links the paracone and protocone. The protocone is damaged, but it appears to be a rounded cusp, and it is connected to the anterolabial cingulum by a short preprotocrista. Stylar cusp D is positioned anterobucally to the metacone, and these two cusps are connected by an anterobuccally-orientated, ridge-like premetacrista. A crest extends posteriorly from stylar cusp D to the posterior margin of the tooth before turning lingually to form the posterior cingulum. The metaconular hypocone is damaged, but was evidently connected to the posterior cingulum via the posmetaconulecrista. The metacone and metaconule are linked via a strongly developed transverse crest.

The M3 is similar in overall morphology to M2, but appears more strongly bilophodont: stylar cusp C is transversely in line with the paracone and protocone, with all three cusps joined by a strongly developed crest; likewise, stylar cusp D, the metacone and metaconule are aligned transversely and connected by a crest. The anterolabial and posterior cingula are relatively well-developed.

The M4 is similar in overall morphology to M3, but is smaller and has less prominent cusps, particularly the metaconule. A transverse crest linking the paracone and protocone is still present, and a very small valley is present between this crest and the anterolabial cingulum, which curves around from the buccal margin of the paracone and merges with the preprotocrista that extends from the apex of the protocone. A similar transverse crest extends lingually from the metacone, but it does not contact the reduced metaconule. A moderately well-developed posterior cingulum is present that merges lingually with the postmetaconulecrista, and terminates buccally behind the metacone.

*Cranium*

Total preserved length of the skull is approximately 197 mm. The dorsal part of the cranium is not preserved. Viewed ventrally, the cranium is preserved in roughly two planes. The more ventral plane comprises the right premaxilla, cheekteeth, zygomatic arch and partial palate; the more dorsal plane comprises the remainder of the preserved cranium. The total displacement between the two planes is approximately 40mm.

The palate is partially preserved but split between the two planes of preservation. A palatal vacuity is identifiable on the left hand side, with its anterior margin level with the anteriormost part of M3; this vacuity appears to be fully enclosed by the palatine, and so is a “palatine fenestra” sensu Voss and Jansa^7,8^. In marsupials that have only a single pair of palatal vacuities, these are typically between the maxilla and palatine, i.e. a “maxillopalatine fenestra” *sensu* Voss and Jansa^7,8^; among vombatiforms, this is seen in wynyardiids, thylacoleonids, fossil phascolarctids and the vombatid *Nimbavombatus*. Presence of palatine vacuities only is seen in *Mukupirna*, the extant phascolarctid *Phascolarctos* and most vombatids (pers. obv.).

The ventral portion of the right maxilla is relatively well preserved, but the suture with premaxilla is not identifiable. The right masseteric process appears weakly developed, although this might be a result of damage. In lateral view, a shallow fossa appears to be present on the lateral surface of the jugal. Most vombatids (but not *Warendja* or *Nimbavombatus*)^9-11^ have a much larger fossa in this region, extending across the maxilla and jugal; in extant wombats, it houses a greatly enlarged superficial masseter^11-13^. A small fossa is also found in this region in the wynyardiids *Muramura* and *Namilamadeta*, although it is unclear whether this represents a precursor to the morphology seen in vombatids (i.e. associated with the superficial masseter), or whether it reflects enlarged snout musculature^2,6,14,15^. Based on available evidence, we are uncertain as to its function in *Mukupirna*.

The right zygomatic arch is partially preserved: in ventral view, it originates anteriorly level with the anterior margin of M1, extending posteriorly more or less parallel with the toothrow. In lateral view, it appears quite shallow, although its exact depth is difficult to determine due to breakage. Posteriorly, the zygomatic arch is broken at a point approximately level with the pterygoids; however the posterior part is partially preserved on the more dorsal plane. The suture between the jugal and the squamosal is impossible to determine. The glenoid fossa appears broad mediolaterally. Unlike the glenoid fossa of most diprotodontians, it appears planar, without a distinct raised articular eminence (anteriorly) or groove-like mandibular fossa (posteriorly)^16,17^. As preserved, the glenoid fossa is not enclosed posteriorly by a raised postglenoid process. A slightly rugose area at the back of the glenoid fossa might represent the remnant of a postglenoid process that has broken away, or alternatively an attachment area for ligaments; if a postglenoid process was present, it would probably have been weakly developed. A shallow squamosal epitympanic fossa or sinus appears to be present posteromedial to this area.

The suture between the alisphenoid and squamosal is not preserved, and it is not possible to determine which of these bones contributed to the roof and floor of the tympanic cavity. The right pterygoid is partially preserved, and is relatively large. The right entocarotid foramen is also identifiable. The basisphenoid is poorly preserved. Further posteriorly, the occipital condyles of the basioccipital are large, rounded and laterally flaring. The jugular foramen is prominent; a forman posterior to this, close to the lateral edge of the basioccipital, is probably a hypoglossal foramen, but it is unclear whether or not there were two hypoglossal foramina on each side (as is usual for marsupials).

*Postcranial skeleton - general*

Postcranial elements preserved in specimen AMNH FM 102646 are as follows: right and left scapulae; left humerus; left ulna; fragment of left radius; left pisiform; left scaphoid; left capitatum (= magnum); left metacarpals I, III, IV and V; right metacarpals II and IV; three proximal and two medial (intermediate) phalanges, and two distal (ungual) phalanges, tentatively interpreted as belonging to digits III (proximal, medial and distal phalanges), IV (proximal phalanx only) and V (proximal, medial and distal phalanges) of the left manus; left and right femora; left tibia (broken distally); left fibula; left calcaneum; left astragalus; left cuboid; left navicular; left ectocuneiform; left entocuneiform; four partial ribs; four caudal vertebrae.

*Scapula*

Both right and left scapulae are preserved and nearly complete; fortunately, most regions missing from the right scapula are preserved on the left side, and vice versa, permitting a relatively complete description. The overall outline is broadly similar to that seen in *Phascolarctos* and *Ilaria*^18^. The scapula is short and robust, measuring 15cm from the supraglenoid tubercle to the dorsal end of scapular spine, and 7.35cm at its widest point. The glenoid cavity is topped by a robust supraglenoid tubercle that extends ventrally. This tubercle has a short, robust coracoid process, which extends ventromedially. Although apparently broken, it was probably not as medially extensive as in, for example, *Phascolarctos* and *Trichosurus* (where it serves for attachment of the coracobrachialis muscle, and probably reflects their capacity to climb)^19,20^. There is an enlarged area on the caudal edge of the scapular blade, just dorsal to the glenoid cavity, for attachment of the caput longum of the triceps brachii muscle; this is also seen in other large, terrestrial vombatiforms, but not *Phascolarctos*^18^. The scapular spine is thick, and the acromion at its ventral end is preserved. The spine is oriented roughly perpendicular to the main blade, and reaches its greatest height at the neck. As in *Ilaria*^18^, the lateral edge of the spine protrudes caudally quite considerably . Another highly distinctive feature shared with *Ilaria* is the marked anterior curvature of the scapular spine as it extends dorsally; in other vombatiforms, the scapular spine is more-or-less straight^18,21^. The neck of the scapula is strongly curved cranially, but markedly less so caudally, where the margin of the scapula is almost straight (this is more evident in left scapula). As a result, the cranial border of the scapular blade bulges out at the approximate midpoint of the scapula, resulting in a distinctly concave outline for the ventral part of the cranial border and a prominent scapular notch; the degree of concavity is more than in *Phascolarctos*^21^, but less than in *Ilaria*^18^. The scapular spine follows the curve of the bulge in the cranial border (see above), as in *Ilaria*^18^. The subscapular surface is somewhat obscured by plaster on both scapulae, but it can be seen to curve medially, as in wombats and *Ilaria*^18^.

*Humerus*

The left humerus is relatively well-preserved, but it has not been fully prepared out of its plaster jacket, and so only the cranial aspect is fully visible. Relative to the size of the cranium of AMNH FM 102646, the humerus appears short, stout, and wide distally due to the presence of prominent epicondyles. Maximum preserved length of the humerus is 17.9 cm; maximum width at its distal end is 7.7 cm; minimum circumference at its midpoint is 13.3 cm (this includes plaster jacket, and so is a slight overestimate); minimum circumference at its distal end is 10.8 cm (this includes plaster jacket, and so is a slight overestimate). The proximal end is damaged, but the greater tubercle appears large and relatively extensive laterally, although not markedly taller (proximally) than the lesser tubercle. The shaft is relatively straight. A prominent deltopectoral crest extends distally from the greater turbercle, reaching its highest point at the approximate midpoint of the humerus. The deltopectoral crest is relatively elongate (10.8 cm), giving a Shoulder Moment Index (SMI; = deltopectoral crest length/humeral length) of 0.6, which is slightly greater than that of the living wombats *Vombatus ursinus* and *Lasiorhinus latifrons*, but less than that of *Palorchestes azael* and *Phascolonus gigas*.

Unlike in vombatids, the deltopectoral crest does not overhang the lateral edge of the humerus^9,11,18^. There also does not appear to be a distinct deltoid tuberosity, separate from the deltopectoral crest (unlike in vombatids^9,11^), but the distal end of this crest is damaged, and so this is not certain. Just distal to the distal end of the deltopectoral crest, the entepicondylar bridge flares out distomedially. Although filled with matrix, the supracondylar foramen is clearly identifiable and is oval in outline. The ectepicondylar (lateral epicondylar) crest originates at a point level with the distal end of the deltoid crest, projecting sharply laterally, although its true distal extent and shape (for example, whether it was distinctly hooked distally or not) is unclear due to damage. The ectepicondyle (lateral epicondyle) curves around the lateral edge of the distal humerus, to join the capitulum. Above the capitulum sits a moderately-developed coronoid fossa. The trochlea is poorly preserved, but can be identified extending distally, forming the distalmost part of the humerus. Medial to the trochlea, the entepicondylar (medial epicondylar) crest curves around the entepicondylar bridge and marking its lateral edge. The Epicondylar Index (distal humeral width/humeral length) is 0.44, which is similar to that of the living wombats *Vombatus ursinus* (0.45) and *Lasiorhinus latifrons* (0.44), but less than that of *Palorchestes azael* (0.61) and *Phascolonus gigas* (0.53).

*Ulna*

The left ulna is well-preserved and nearly complete, lacking only the distal epiphysis. The ulna relatively short and robust, with a total preserved length of 20.9 cm. The shaft is slightly curved, such that it is convex laterally and concave medially, as in vombatids, *Ilaria* and *Ngapakaldia*^18^, whereas *Nimbadon* and *Phascolarctos* show a slightly lateral curve towards the distal end^22^. The olecranon (measured from the distal margin of the trochlear notch, as in Hopkins and Davis^23^) is 6.2 cm long, giving an Index of Fossorial Ability (IFA; = olecranon length/(total ulnar length-olecranon length)) of 0.42. This is greater than that of *Ilaria* (~0.31), similar to that of *Vombatus* (0.42) but less than that of *Lasiorhinus* (0.58), *Palorchestes* (0.46) and *Phascolonus* (0.73). The olecranon does not appear as sharply inflected medially as in vombatids.

The anconeal process is prominent, but not as enlarged as in vombatids. The very large anconeal process of modern wombats may prevent disarticulation of the elbow joint when extended during digging^18^; its large size in *Mukupirna* may therefore be an indication of scratch-digging behaviour. The morphology of the anconeal process is unknown in the diprotodontid *Ngapakadia* and ilariid *Ilaria*^18^, but it is small in the living koala *Phascolarctos* and the arboreal diprotodontid *Nimbadon*^22^.

The trochlear notch has three articular facets. The large medial humeroulnar facet is narrow at its proximal end, but widens out distally into a large, flat and shallow basin. This basin projects medially much more than laterally. Dorsolateral to the medial humeroulnar facet is the dorsally-projecting radial humeroulnar facet. This facet is flattened laterally, but it has a well-developed lateral proximal trochlear crest, which extends medially over the medial humeroulnar facet. Distal to the radial humeroulnar facet is a triangular, concave proximal radioulnar facet (radioulnar notch), which is also flattened laterally. The radioulnar facet is buttressed medially by a thick, well-developed coronoid process. In medial view, the coronoid process can be seen extending far above the height of the trochlear notch; it is much taller than in *Ngapakaldia*, but slightly shorter than in *Ilaria* and vombatids^18^. The coronoid process prevents disarticulation of the elbow when flexed, and its large size in *Mukupirna* may indicate that the forelimb transmitted high loads; if so, this may (along with the large anconeal process) be another indication of fossorial or scratch-digging behaviour. A deep, anteroposteriorly extensive fossa for the flexor digitorum profundus muscle is visible in medial view, below the trochlear notch and extending proximal and distal from it. In lateral view, a shallow, short fossa for the anconeus is present distal to the proximal radioulnar facet. Posterior to this fossa, a ridge begins which extends distally, becoming more pronounced and becoming confluent with the anterior edge of the ulna. Posterior to this crest a shallow but wide fossa is present, extending distally to a point approximately two-thirds of the way down the length of the bone.

*Radius*

A fragment of the left radius is preserved. It is broken proximal to the radial tuberosity and distal to a point probably around the midpoint of the complete bone. The preserved fragment is 72.1 mm long, relatively straight and with a minimum width of 12.2 mm. The shaft is roughly cylindrical proximally, becomes more triangular in cross-section distally. The radial tuberosity (for attachment of the M. biceps brachii) is mediolaterally broad, similar in width to the radial shaft. It is broken at its proximal end.

*Pisiform*

This bone is slightly narrower than that of *Ngpakaldia*^18^, but is relatively wider mediolaterally than in vombatids: maximum anteroposterior length is 21.2 mm; minimum width is 10.6 mm. The area of the ulnar facet is greater than that of the cuneiform facet. Collectively, the comparatively wide morphology of the pisiform and the large size of its ulnar facet would suggest terrestrial locomotion and/or the transfer of relatively high forces through the manus^18: 22^.

*Capitatum*

The capitatum (= magnum) is larger and longer than in *Ngpakaldia*, with a longer, more sinuous scapholunar facet; the trapezoid facet is also more concave and better defined than in *Ngapakaldia*^18,24^.

*Scaphoid*

Vombatiforms typically lack a separate lunatum, and this absence has usually been attributed to fusion between the lunatum and scaphoid^25^. However, embryological evidence suggests that the lunatum actually fuses to the distolateral tip of the radius in *Phascolarctos cinereus*^26^ and possibly also in *Vombatus ursinus*^25^, and possible homologues of the lunatum have been identified a few specimens of *Vombatus ursinus*^27^ and *Lasiorhinus latifrons*^24^. Because of this, we refer to the scaphoid here, rather than the “scapholunar” (*contra* Munson^18^). AMNH FM 102646 does not include any bone that could be identified as a homologue of the lunatum, suggesting that *Mukupirna* lacked a separate lunatum, as is typical for vombatiforms^24^; however, the possibility that this bone was originally present but has been lost during fossilisation or collection cannot be ruled out. The scaphoid is more elongate and slightly thinner than that of *Ngapakaldia*. The distal surface of the scaphoid is divided into two distinct facets, an apparently apomorphic condition present in all vombatiforms described to date^24^.

*Metacarpals*

MCI is shorter than the other preserved metacarpals. It is much longer and more slender than that of *Ngapakaldia*, but is very similar in overall morphology to that of *Ilaria*^18^. The proximal facet is concave dorsoventrally and convex mediolaterally, and approximately subtriangular in outline. As in *Nimbadon*^22^, the distal facet for articulation with the proximal phalanx is asymmetrical. The lateral condyle in ventrally orientated and reduced. It sits closer to the central condyle than does the medial condyle. The central condyle is the best developed of the three, and is ventrally directed. The medial condyle is ventromedially directed, and more extensive proximally than the other two condyles.

MCII is more slender than the equivalent bone in *Ngapakaldia*. The proximal end is not expanded, in contrast, the proximal end of MCII is expanded and concave in *Ilaria* and vombatids^18^. The trapezoid facet faces proximomedially, as it does in both *Ngapakaldia* and *Trichosurus*, whereas this facet faces more directly proximally in *Ilaria* and vombatids^18^. The capitatum (= magnum) facet is distinct and much better developed than in *Ngapakaldia*, similar to the morphology seen in *Ilaria* and vombatids, although it does not project laterally as far as it does in the latter two taxa ^18^. The (medial) MCI facet is less proximodistally orientated and has a larger surface area than the (lateral) MCIII facet, and has a larger surface area.

MCIII is the largest metacarpal. The hamatum (= unciform) facet shows a prominent lateral overhang; when articulated, this overhang overlaps onto MCIV almost exactly as in *Vombatus*. The proximal surface is largely convex, but there is a slightly concave dip medially, setting the narrow, rectangular MCII facet off from that of the capitatum; by contrast, in *Ilaria* and vombatids, the MCII and capitatum facets are continuous^18^. The capitatum facet itself is convex, resembling that of *Ilaria* and vombatids; however, this facet is more triangular in *Mukupirna*, whereas it is more semi-circular in *Ilaria* and vombatids^18^. MCIII morphology in *Ngapakaldia* and *Trichosurus* is very different: the capitatum surface is strongly concave, and the proximal end is not expanded, such that the MCII and hamatum facets are nearly flush with the shaft of the bone. In *Mukupirna*, the MCIV facet is large and concave, with two pits present proximoventral to it, and the dorsal surface of MCIII has a strongly developed ridge running from its proximal end to approximately midway along its length.

MCIV has a square proximal articular surface, but the centre of the surface is slightly concave, such that the hamatum and capitatum facets are distinguishable. The MCIII facet faces proximomedially rather than medially. MCIV is slightly longer than MCII, in contrast to the condition in vombatids, in which MCIV is shorter than MCII.

The distal condyles of MCs II, III and IV are all roughly symmetrical and spherically shaped, in contrast to the broad, dorsventrally-flattened morphology present in *Ilaria*, *Phascolarctos* and vombatids^18^. The central condyle is the most prominent and best developed.

MCV is relatively long, being only slightly shorter than MCIV, whereas it is much shorter than MCIV in *Ngapakaldia* and vombatids^18^. Otherwise, it is broadly similar in shape to that of *Lasiorhinus*. The lateral condyle is mediolaterally compressed, but proximodistally extended compared to the others. The central condyle is relatively well developed, more so medially than laterally. The medial condyle is poorly developed and present on the medial edge of the distomedial margin of MCV. The proximal facet is divided into two, with a large, rectangular hamatum facet orientated mediolaterally, and a much more proximolaterally-orientated MCIV facet.

*Phalanges*

Three proximal, two medial (intermediate) and two distal (ungual) phalanges preserved. We tentatively identify them as belonging to digits III (proximal, medial and distal phalanges), IV (proximal phalanx only) and V (proximal, medial and distal phalanges) of the left manus. Overall, they are very similar in morphology to those of *Ilaria* and vombatids, and are indicative of probable fossorial behaviour. They are much shorter and smaller than in *Ngapakaldia*, with less strongly-developed proximal and distal condyles. They are dorsoventrally flattened, with the distal ends of both the proximal and medial phalanges dorsoventrally tapered; the degree of tapering is somewhat less than in *Ilaria* or vombatids, but more so than in *Ngapakaldia*, which lacks any such tapering^18^.

On the palmar surface of each proximal phalanx, two weakly developed palmar tuberosities are clearly identifiable. The proximal articular facet is concave mediolaterally and dorsoventrally. The distal condyles are flattened dorsoventrally and weakly saddle-shaped. Proximal phalanx V exhibits a medial twist of its distal end and lateral buttressing of the proximal end, as seen in vombatids. This twist serves to bring digit V closer to the others, rather than in a more “splayed” position seen in *Ngapakaldia*, and in living wombats allows the manual digits to form a shovel-like structure for digging.

The two medial phalanges have rounded and saddle-shaped medial and lateral condyles, much more so than those of the proximal phalanges. The proximal articular facets are smooth and distinctly kidney-shaped.

The two distal phalanges are relatively long (maximum length of 24.57 mm and 20.4 mm) compared with those of *Ngapakaldia*. They are not as deep dorsoventrally, nor compressed mediolaterally as those of *Ngapakaldia*, but instead appear distinctly dorsoventrally flattened, as in *Ilaria* and vombatids^18^. The articular facets are moderately to deeply concave, with large flexor tubercles.

*Femur*

The left and right femora are equally well-preserved. The left femur is 23.0 cm long, with a minimum circumference of 8.35 cm at its midpoint. The right femur is somewhat flattened anterioposteriorly, and is 22.5 cm long, with a minimum circumference of 8.5 cm at its midpoint. It is similar to that of *Vombatus* in overall morphology, but markedly larger (mean femoral length is 15.6 cm in *Vombatus ursinus*)^28^. Overall the proximal portion of the femur is wide and inclined medially. The femoral head is crushed and poorly preserved in both specimens, but appears to be somewhat elongate, as in vombatids. The base of the greater trochanter flares out markedly laterally at a point approximately 10cm from the top of the femur. The top of the greater trochanter is approximately level with the head of the femur. The femoral trochantic fossa is deeply incisive; however, the opening of the fossa is wide and rounded, as opposed to narrow and slit-like in *Wynyardia*^29^. The femoral trochantic fossa does not extend far ventrally; however, it extends further in the left femur, to about the midpoint of the lesser trochanter, as opposed to the top of the lesser trochanter in the right femur. The lesser trochanter is badly preserved on the left femur and is missing from the right. A well-developed third trochanter (a rugose flange of bone for attachment of the gluteal muscles) is present distal to the major trochanter; presence of a large third trochanter in notoryctid marsupial moles has been proposed to reflect digging behaviour^30-32^. A prominent third trochanter is also present in vombatids, but it is absent in other vombatiforms, including *Ngapakaldia* and *Wynyardia* (the femur is currently unknown for *Ilaria*)^18,29^, and it is rare among marsupials generally^33^. The shaft is straight with a largely flattened posterior surface. The distal femur is relatively well preserved on both sides. A depression is present beneath the medial condyle, similar to the morphology seen in *Wynyardia* but somewhat smaller. As in *Wynyardia*, the medial condyle is slightly larger than the lateral condyle^29^. In anterior view the medial condyle is more displaced medially than in *Wynyardia*, but this could be a result of post-depositional deformation.

*Tibia*

Only the left tibia is preserved: it is badly crushed and deformed, with the distal end largely broken away. The distal condyle is preserved separately. The major preserved fragment is 15.4cm long, and when intact was probably shorter than the femur, as in all vombatiforms^22^. The proximal end is relatively poorly preserved and appears displaced medioventrally with respect to the shaft of the tibia, but a number of features can be identified. The intercondylar eminence is a rounded knob. The medial femorotibial facet is concave, with a crushed anterior portion. The lateral femorotibial facet is convex. As in *Wynyardia*, a pronounced tibial crest is present, but the exact extent and orientation of this crest is impossible to determine due to crushing and distortion. The distal end is well preserved and relatively complete, and is only missing the posterior part of the lateral astragalotibial facet. The preserved part of this facet is wide and broad, occupying approximately two thirds of the distal end; it is not as roundly convex as in *Vombatus*. The medial malleolus (medial astragalotibial facet) occupies the remaining third. The medial malleolus is narrower anteriorly and expands posteriorly; it is much larger than in *Ngapakaldia*^18^. As in vombatids and *Ngapakaldia*, there is a deep pit, presumably for ligamentous attachement, posterolateral to the medial malleolus; Munson^18^ argued that these ligaments act to limit extension of the foot.

*Fibula*

The left fibula is somewhat crushed, but the shaft is relatively well-preserved; the distal epiphysis is preserved separately. Total length is 17cm. A parafibula (= fibular fabella) is present. The proximal end of the fibula is damaged, and so it is unclear whether it was mediolaterally expanded to the extent seen in most other vombatiforms. The lateral notch or peroneal groove at the distal end of the fibula (which houses the digital flexors) is much deeper than in *Ngapakaldia* or vombatids^18^. The distal epiphysis of the fibular preserves facets for contact with both the astragalus and the calcaneus; these facets are relatively flat and approximately heart-shaped. In distal view, the calcaneal facet projecting slightly more distally than the astragalar facet in distal view.

*Astragalus*

In dorsal view, the astragalus is roughly triangular in shape, with a rounded navicular facet. The fibular facet is triangular and flat. The lateral tibial facet is relatively broad and well-trochleated. The ridge between the medial and lateral tibial facets (the “tibial knob” *sensu* Munson^18^), is rounded and not well-defined. The medial tibial facet is prominent, as in vombatids. In ventral view, the prominent medial plantar tuberosity is almost ball-like, while the sustentacular facet is convex, and is continuous with the ectal facet. Distally, the sustentacular facet is connected to the navicular facet via a distinct facet for contact with the cuboid, as in other vombatiforms^34^. The ectal facet is slightly more concave than it is in *Vombatus*. Overall, the astragalus is larger and more robust than that of *Ngapakaldia*, with better defined facets^18^.

*Calcaneus*

The calcaneus is a short, robust element, similar to that of *Ngapakaldia* but somewhat more gracile and straighter overall^18^. The posterior epiphysis has not fully fused to the main body of the calcaneus. The calcaneal tuber is quite short with respect to the head. The calcaneocuboid facet sits at an oblique angle relative to the anteroposterior axis of the calcaneus. The distal calcaneoastragalar contact is proximodistally short but mediolaterally broad (broader than in *Ngapakaldia*). There is a distinct distal extension of the sustentacular facet, as in other vombatiforms, and overall the sustentacular facet appears more elongate proximodistally than that of vombatids^34^.

*Navicular*

In overall shape, the navicular resembles that of *Ngakapaldia*, except that the ventrolateral bulge is more pronounced and the astragalar facet is more oval, as in *Vombatus*. There appears to have been broad contact between the navicular and the mesocuneiform, as in vombatids and *Ngapakaldia* *bonythoni*, whereas this contact is somewhat narrower in *N. tedfordi* and *Phascolarctos*^18,34^.

*Cuboid*

The cuboid resembles that of *Ngapakaldia*^18^, except that the fossa on the calcaneal facet is more deeply developed and there is a more strongly pronounced dorsal lip at its dorsal extremity. The distal facet as a whole is also not as dorsoventrally compressed as in either *Ngapakaldia* or vombatids, although the width of the facet is still greater than its height. There also appears to have been greater contact with the astragalus than in either *Ngapakaldia* or vombatids. The facets for MTIV and V are broad but not strongly differentiated. These facets are strongly inclined posterolaterally-anteromedially in dorsal view.

*Entocuneiform*

In overall morphology, the entocuneiform strongly resembles that of *Ngapakaldia*, except that is more robust with a broader, thicker posterior section^18^; however, it differs from *Ngapakaldia* and more closely resembles vombatids in having a broader navicular facet. The MTI facet is more convex than in *Ngapakaldia* and is more like that of vombatids in this regard.

*Ectocuneiform*

The ectocuneiform is roughly square in dorsal outline, resembling the morphology seen in *Ngapakaldia*, but unlike that of vombatids, in which this bone is wider than long^18^. However, the morphology of the ectocuneiform facets are more like the morphology seen in *Vombatus*. The sharp (rather than rounded) dorsal border of the cuboid facet would result in a tighter fit between these two bones than in *Ngapakaldia*. The broader, flatter contacts for both the navicular and mesocuneiforms are also more like those of vombatids than *Ngapakaldia*. The plantar process is also less strongly developed than in *Ngapakaldia*. The MTIII facet is rectangular.

*Ribs*

Four partial ribs are preserved, but are of limited functional and phylogenetic informativeness.

*Caudal vertebrae*

Three distal caudal and one proximal caudal vertebrae are preserved. The distal caudal vertebrae are saddle-shaped, short and squat, with few processes preserved. The proximal caudal vertebra has a short, saddle-shaped body, with short, posterodorsally-directed transverse processes; however, most of the neural spine and articular processes are broken and only partially preserved. Based on these four vertebrae, it is unclear how exactly how long the tail was; however, the centra are relatively longer than those in vombatids, suggesting that the tail as a whole was also somewhat longer (*Vombatus ursinus* has only 11 caudal vertebrae).

**Relevance of *Marada arcanum***

*Marada arcanum* is an enigmatic vombatiform that was described based on a single right dentary from Hiatus site at Riversleigh, and referred to its own family, Maradidae.^35^ Hiatus site is part of Riversleigh Faunal Zone A, which is currently interpreted as late Oligocene in age^36,37^, i.e. approximately coeval with the Pinpa Local Fauna. A single left lower molar (NTM P2815-11) from the Pwerte Marnte Marnte Local Fauna in the Northern Territory (which is probably also late Oligocene in age) may also represent *Ma. arcanum*, or another member of the same family{Murray, 2006 #526}{Black, 2007 #4537}. The lower molars of *Ma. arcanum* are bunolophodont, but they retain a distinct (albeit weak) paracristid, postmetacristid, preentocristid and cristid obliqua, as in selenodont taxa. They show greatest overall similarity to the lower molars of wynyardiids, and we have discussed the clear similarity between the upper molars of *Mukupirna* and those of wynyardiids. The lengths of the postcanine dental series of holotypes of *Mu. nambensis* (53 mm) and *Ma. arcanum* (51 mm) are also very similar. It is therefore possible that *Mu. nambensis* is in fact *Marada arcanum* or a closely related taxon. However, upper molars of *Mukupirna nambensis* have not been found at Riversleigh, nor have lower molars of *Marada arcanum* been reported from the Pinpa Local Fauna. Only the discovery of associated upper and lower dentitions of either *Mu. nambensis* or *Ma. arcanum* will resolve this issue.

**List of morphological characters**

1. Number of upper incisors (ordered):

0. Five

1. Four

2. Three

3. One

2. First upper incisor:

0. Not greatly broadened mediolaterally

1. Greatly broadened and "straplike"

3. Upper canine:

0. Present

1. Absent

4. P1:

0. Present

1. Absent

5. P2:

0. Present

1. Absent

6. P3:

0. Premolariform

1. Semi-sectorial

2. Sectorial

3. Molariform

4. Bicuspid

7. Bladelike P3 - ratio of maximum length to maximum width:

0. <1.5

1. >1.5

8. Bladelike P3:

0. Ridged

1. Smooth-sided

9. Posterobuccal cusp on P3:

0. Absent

1. Present

10. Prominent lingual cusp on P3:

0. Absent

1. Present

11. Enamel extending down the buccal surface of P3 and onto the root:

0. Absent

1. Present

12. Enamel tracts extending down lingual surface of upper molars and buccal surface of lower molars:

0. Absent

1. Present

13. Hypselodont cheek teeth:

0. Absent

1. Present

14. Molar shape:

0. Not strongly bilobed

1. Strongly bilobed

15. Enamel crenulations:

0. Absent/weak

1. Strong, crest-like

16. Selenodonty/distinct centrocrista (ordered):

0. Absent

1. Weakly developed

2. Strongly developed

17. Lophodonty (ordered):

0. Absent

1. Present but weakly developed

2. Present and strongly developed - individual cusps no longer identifiable

18. Fully lophodont molars:

0. Without midlink

1. With midlink

19. Anteriorly concave lower / anteriorly convex upper molar lophs:

0. Absent

1. Present

20. Metaconule:

0. Weakly developed - tooth triangular

1. Enlarged, forming posterolingual cusp - molars square

21. M1 paraconule:

0. Absent/weak

1. Moderate/strong

22. Neometaconule on M1:

0. Absent

1. Present

23. Protostyle:

0. Absent

1. Present

24. Neomorphic cuspule at base of metaconule of M1:

0. Absent

1. Present

25. Posterolingual paracristae:

0. Absent/weak

1. Strongly developed

26. Postprotocrista:

0. Present

1. Absent

27. M4 metaconule:

0. Absent/ significantly reduced and retracted towards posterior cingulum

1. Distinct, cuspate

28. Posteriorly increasing molar gradient (ordered):

0. M4 absent

1. M4 markedly smaller than M1

2. Absent, M4 and M1 similar in size, or M4 slightly smaller

3. Present, M1 clearly larger than M4

29. Number of lower incisors (ordered):

0. Four

1. Three

2. Two

3. One

30. Anterior lower incisor:

0. Not greatly enlarged

1. Greatly enlarged and procumbent

31. Dorsal surface of procumbent incisor:

0. Not strongly concave

1. Strongly concave, shovel-shaped

32. M1 metaconid:

0. Present

1. Absent

33. Apex of metaconid is anterior to apex of protoconid on m2-3:

0. No

1. Yes

34. Protostylid on m1:

0. Absent

1. Present, cusplike

2. Present, vertically directed crest

35. Entostylid ridge on m1:

0. Absent

1. Present

36. Metastylid on lower molars (ordered):

0. Absent

1. Present, cuspate

2. Present, metastylid fold

37. Cristid obliqua:

0. Present, well-developed

1. Absent or indistinct

38. Distinct and separate paraconid on m2-3:

0. No

1. Yes

39. Termination of m2-3 paracristid:

0. Terminates in lingual position, anterior to metaconid

1. Terminates in more labial position, anterolabial to metaconid

40. Presence of a crest connecting entoconid and hypoconid:

0. Absent

1. Present

41. Incisive foramen in deep fossa formed by intradiastemal ridges/crests that "V" posteriorly to a point at or posterior to incisivomaxillary suture:

0. No

1. Yes

42. Incisive foramen in ovate fossa:

0. No

1. Yes

43. Incisive foramen located posteriorly:

0. No

1. Yes, the incisive foramina lie posteriorly, just anterior to P3, in a deep pocket at the rear of the diastema - the posterior position of the foramina correspondingly displaces the incisivomaxillary suture to a position just anterior to the cheek tooth row

44. Posterior palatal vacuities (maxillopalatine vacuities) 1:

0. Present

1. Absent

45. Posterior palatal vacuities 2:

0. Between maxilla and palatine

1. Within palatine

46. Nasal aperture retracted beyond incisor arcade (ordered):

0. Absent or just posterior to incisor arcade

1. Retracted to above diastema (or level of incisivomaxillary suture)

2. Retracted to above cheek tooth row

47. Nasals:

0. Not markedly broadened anteriorly

1. Markedly broadened anteriorly

48. Nasomaxillary suture length relative to naso-premaxillary suture length (dorsal view):

0. Nearly same or greater

1. Far less than naso-premaxillary suture length

49. Direction of frontonasal suture from midline to lacrimal bone (dorsal view):

0. Lateral

1. Rostrolateral

50. Nasals contact lacrimals:

0. No

1. Yes

51. Combined breadth of nasal bones in dorsal view:

0. Much less than length

1. Equal to or greater than length

52. Postorbital/supraorbital process of frontal bone in dorsal view (ordered):

0. Small or absent

1. Well-developed

2. Laterally extensive, contacting frontal process of jugal to form complete postorbital bar

53. Postorbital process:

0. Formed by frontal only

1. Formed by frontal and lacrimal

54. Sagittal crest:

0. Absent

1. Present

55. Infraorbital foramen:

0. Round or oval

1. Slitlike

56. Masseteric process; ventral extent (ordered):

0. Absent or indistinct

1. Short

2. Elongate

57. Masseteric process: lateral extent:

0. Not markedly expanded laterally

1. Greatly expanded laterally

58. Masseteric process composition:

0. Maxilla and jugal

1. Jugal only

59. Presence of a single and extensive area for attachment of masseter muscles extending from anterior to orbit to beneath orbit and traverses both maxillary and jugal bones:

0. Absent or indistinct

1. Forming prominent depression

60. Lacrimal tubercle/tuberosity:

0. Absent

1. Present

61. Palatine-lacrimal contact in orbit:

0. Present

1. Absent - frontal-maxilla contact

62. Frontal-squamosal contact:

0. Absent, alisphenoid-parietal contact

1. Present

63. Glenoid fossa 1:

0. Articular eminence planar or concave, mandibular fossa absent or indistinct

1. Articular eminence planar, mandibular fossa well-developed

2. Articular eminence strongly convex and broad

64. Postglenoid process (ordered):

0. Absent or weakly developed

1. Present, vertical

2. Present, ventral edge curving posteriorly to partiallyfloor external auditory meatus

65. Position of postglenoid foramen:

0. Posterior to postglenoid process and bounded medially by petrosal

1. Anteromedial to or in line with postglenoid process

2. Posteromedial to postglenoid process within squamosal

3. Within epitympanic fenestra, surrounded by bony septum

66. Tympanic cavity roof elements (ordered):

0. Alisphenoid (no squamosal contribution)

1. Alisphenoid and squamosal

2. Squamosal (no alisphenoid contribution)

67. Tympanic floor elements (ordered):

0. Alisphenoid

1. Alisphenoid and squamosal

2. Squamosal

68. Tympanic wing, whether squamosal or alisphenoid (ordered):

0. Absent

1. Does not contact exoccipital

2. Contacts exoccipital

69. Epitympanic fenestra developed in postglenoid cavity:

0. Absent

1. Present

70. Non-auditory sinuses:

0. Absent

1. Present

71. Posterior epitympanic sinus:

0. Absent / weak

1. Moderate to deep

72. Interparietal:

0. Present, large

1. Absent, very small or polymorphic

73. Ventrolaterally flared mastoid process on occiput:

0. Absent

1. Present

74. Size of paroccipital processes:

0. Small

1. Large

75. Posterior extent of mandibular symphysis on mandible (ordered):

0. Anterior to p3

1. Below p3

2. Below m1

3. Below m2-3

76. Flared masseteric eminences on mandible:

0. Absent/weak

1. Moderately to strongly flared

77. Masseteric foramen:

0. Absent

1. Present

78. Fused mandibular symphysis:

0. Absent

1. Present

79. Subarcuate fossa:

0. Deep and well-excavated

1. Shallow depression

80. Humerus, deltoid ridge:

0. Has no lateral overhang, or a very minor one

1. Has a large flaring overhang

81. Cuneiform, position relative to scapholunar:

0. Directly lateral to scapholunar

1. More proximal than the scapholunar

82. Cuneiform, ridge between ulnar and pisiform facets:

0. Middle section sinks below the rim of the cuneiform

1. High rising throughout, straight

83. Scaphoid, distal surface:

0. Single facet, smooth

1. Separated into two distinct facets

84. Scaphoid, distolateral process:

0. Absent

1. Present

85. Unciform, hamate process:

0. Large, hooked and curves medially

1. Reduced, not as hooked and does not curve as far medially

86. MC II, proximal end:

0. Mediolaterally compressed

1. Expanded and concave

87. MC III, facet for MC IV:

0. Proximal only to ligament pit (no dorsal component)

1. Dorsal to ligament pit (no proximal component)

2. Dorsal and proximal to ligament pit

88. MC III, facet for MC IV:

0. Facing laterally or only slightly distally slanted

1. Strongly distally slanted or completely facing distally.

89. MC III, magnum facet:

0. Grooved or concave

1. Convex or flat to slightly convex

90. MC III, MC II facet:

0. Separate, distinct, and lateral to the magnum facet

1. Continuous with the magnum facet

91. Proximal phalanges, manus or pes, distal ends:

0. Not tapered

1. Dorsoventrally tapered, so that the articulation for the medial phalanx faces ventrally

92. Pelvis, ilium:

0. Only slightly dorsoventrally flattened, or mediolaterally flattened

1. Greatly dorsoventrally flattened

93. Fibula, lateral notch on distal end:

0. Clearly present

1. Extremely slight or absent

94. Fibula, femoral facet

0. Not present

1. Present

95. Astragalus, lateral tibial facet:

0. Mostly convex

1. Mostly concave

96. Astragalus, head:

0. Wider than long

1. Narrow, mediolaterally compressed, or length and width are equal

97. Cuboid:

0. No contact

1. Contacts astragalus

98. Entocuneiform, MT I facet:

0. Saddle-shaped (concavo-convex)

1. Convex

99. Metatarsal I, entocuneiform facet:

0. Mostly convex

1. Strong concave component

**Morphological matrix in NEXUS format**

#NEXUS

Begin data;

Dimensions ntax=42 nchar=99;

Format datatype=standard symbols="012345" gap=-;

Matrix

Didelphis_marsupialis 000000--01000-020--0000-000200-0000001000000000010010100--0000012001000001000000???????????0?11?0??

Perameles_bougainville 000000--010000020--10000000110-0000001000000000010000000--00010100010011000000000001102000000001000

Galadi_speciosus 000000--010000020--10000000110-000000100000000001000-100--000001000100010000000????????????????????

Cercartetus_lepidus 200000--000000001--10000-001?100100000100000000010000000--0000011102001000200000??000????????1000??

Ngapakaldia_spp. 200110--010000002011---0-1133100000010110001-101100001010101?1113211111?012010101110002000??1110101

Kolopsis_torus 201110--010000002011---0-1133100000010110001-1001000010200010111??{01}111??012?01?0????1???????11?????

Neohelos_stirtoni 201110--010000002011---0-1133100000010110001-000100001020001?1113211111?0120101????????????????????

Nimbadon_lavarackorum 201110--010000002011---0-1133100000010110001-000100(01)010200010111321111100120(01)(01)1011101020001?01?????

Silvabestius_spp. 200110--010000002011---0-1123100000010110001-00110010102000100113211111001??1??????????????????????

Litokoala_kutjamarpensis ???11110010000120--111111002???01112001000000????????1?100????111111001001????0????????????????????

Litokoala_dicksmithi 2001110-010000120--111101002????????????0000?00000010?0100010??????????????????????????????????????

Madakoala_spp. 200111?0110000(01)20--111000002310001010010??????????????????????????????????2??0?????????????????????

Nimiokoala_greystanesi 2001110-110000120--111101002310011110010000??00?1?0??101000110???1??0010??20100????????????????????

Perikoala_robustus ???111??110000120--11100000231?00101001(01)???????????????0--????????????????2????????????????????????

Phascolarctos_cinereus 20011100010000120--1111(01)10123100011200100000100100110100--011(01)11110110100121110011100000??0001?????

Priscileo_pitikantensis 2000011100000010???1000?00?1???????????????????????????0--?????????????????????0???????????????????

Priscileo_roskellyae 20000111000000101-01000000?1?101-20000?00000000010010100--000011211100100010100????????????????????

Wakaleo_oldfieldi 200??111000000101-01000000-03101-20000?0??????????????????????????????????1??0?????????????????????

Wakaleo_vanderleuri 20001111000000(01)0???1000000-03101-20000?00000000010010100--00?0112?110?1?011?10?????????????????????

Sedophascolomys_medius 31111?????0111?????1???????231??????????10101?0???????????????????????????3111?????????????????????

Phascolonus_gigas 31111?????0111?????1???????2310?????????1010120????0?010--1???2022-0??1?1?3111?1111?11?1?1111110???

Ramsayia_magna 30111??????111?????1???????2310??????????0?01?0??????????????????????????????1?????????????????????

Lasiorhinus_krefftii 30111?????1111?????1???????2310?????????010011000(01)110000--110(01)2022210011103111?????????????????????

Lasiorhinus_latifrons 30111???001111011-0100000002310?00000111010011000(01)110000--1101202221001110311111111011?11111111011?

Mukupirna_nambensis 20011???001000011-0100000012????????????0?001?????????????0???00????????1??????0??10?011?01?0??????

Vombatus_ursinus 30111?--001111011-01000000?231000000011(01)00001101100(01)0010--11012022210011103111111110111111111110111

Vombatus_hacketti 30111?????1111?????1????????310?????????000011011?000010--11??202?{12}10?111?3111?1???????????????????

Warendja_wakefieldi ?0?11?????1111?????1???????2310????????1??000???1????100--0?1000222100?1??2110?1???????????????????

Muramura_spp. 20011100010000011-01000000123100000000110000000???000102000???112211000?11101010????????????011????

Priscakoala_lucyturnbullae ???????????000020--1000000???????1010??0???????????????????????????????????????????????????????????

Zygomaturus_trilobus 201110--010000002011---0-1133100000010111?01-011??0??1020001??12??0101??01300112?11?10?0?01????????

Diprotodon_notatum 211110--000000002011---0-1133100000010111001-100100000020001?112?????11?00200112111?10?0001?1??????

Euryzygoma_dunense 201110--000000002011---0-1133100000010?11001-11??????102100???1{12}?????1??0??????????????????????????

Propalorchestes_novaculacephalus 201110--010000002111---0-11231?0000000?100?1-2011010010200011011?21{12}111?1121(01)1?????????????????????

Palorchestes_painei 201110--010000002111---0-11331?0000000110001-20?101???0{12}0?0????1??2?1???1120?1?????????????????????

Palorchestes_azael 201110--010000002111---0-1133110000000110001-20?1011100{12}0001112032??111?1130011????????????????????

Namilamadeta_spp. 20011110010000011-010000001231000000001100000(12)00100(01)0102000011112211000?1120101????????????????????

Ilaria_spp. 200110--110000020--10000001331000(01)00000100?0??????????02000???11?2{12}10???1?21011??????111111????????

Kuterintja_ngama ???110--010000020--100000012310000000000???????????????20?????????????????2??0?????????????????????

Thylacoleo_carnifex 20000111000000100--1000???-0{123}101-20?0??00000000110020(01)00--01011232211110000010(01)?11100001100?1??????

Nimbavombatus_boodjamullensis ??0110--001100011-01???0?012?????????????0000?????????00--0?0??????????????????????????????????????

Djarthia_murgonensis ??0000--000000020--110000002???000000100??????????????????????????????????????0???????????????0????

;

End;

**Justification for body mass estimates**

A number of different methods for inferring body mass of fossil mammals have been proposed in the literature. In the case of vombatiforms, applying these consistently is difficult because the various living and extinct taxa differ markedly in relative craniodental and postcranial proportions, reflecting the functional demands of their different ecologies (e.g. herbivory in diprotodontids versus carnivory in thylacoleonids; digging in vombatids versus climbing in *Nimbadon* and *Phascolarctos*). For example, the molars of palorchestids increase in size posteriorly^38-41^, whereas the molars of thylacoleonids decrease sharply in size^42^, with *Thylacoleo carnifex* having lost M2-4 and m3-4^43,44^. Thus, the areas of the fourth upper and lower molars, which Myers^45^ found to be two of the most accurate measurements for inferring body mass for diprotodontians, are unlikely to perform equally well for palorchestids and thylacoleonids, and in fact cannot be used for *Thylacoleo carnifex* as it entirely lacks the relevant teeth. Furthermore, regression equations are usually based on living species of known weight, but most fossil vombatiforms lack obvious ecological analogues, at least within the living Australian marsupial fauna. For these reasons, it seems inappropriate to use a single measurement for inferring body mass of fossil vombatiforms.

For the vombatiform body mass estimates presented here, we used the following criteria, in order of preference: 1) if a recent published estimate was available, we used this, provided it was clearly based on a on well-justified, explicitly quantitative methods; 2) if no recent published estimate was available, but humeral and/or femoral material was known, we calculated an estimate using the regression equations of Richards et al.^46^, which are based on humeral circumference, femoral circumference, and combined humeral and femoral circumference (if at least one humerus and one femur was available for a particular taxon, we used all three of the Richards et al.^46^ equations, and calculated a mean value); 3), where postcranial material was not available, we used the craniodental regressions of Myers^45^, using his “diprotodontians” dataset—for each of these taxa, we used up to four of the highest ranking (as measured by total rank) equations that could be calculated based on available material, incorporating the relevant smearing estimates, and then used these estimates to calculate a mean value.

Taxon: *Didelphis marsupialis*

Body mass: 1134.75 g

Justification: PanTHERIA database^47^

Taxon: *Djarthia murgonensis*

Body mass: 42.3 g

Justification: mean of four different body mass estimates presented by Beck^48^

Taxon: *Perameles bougainville*

Body mass: 230.8 g

Justification: PanTHERIA database^47^

Taxon: *Galadi speciosus*

Body mass: 917.5 g

Justification: Warburton and Travouillon^49^

Taxon: *Cercartetus lepidus*

Body mass: 8.03 g

Justification: PanTHERIA database^47^

Taxon: *Ngapakaldia* spp.

Body mass: 119200 g

Justification: Richards et al.^46^

Taxon: *Kolopsis torus*

Body mass: 156000 g

Justification: Mean of estimates for small and large forms given by Murray et al.^50^

*Neohelos stirtoni*

Body mass: 173300 g

Justification: Mean of estimates from Richards et al.^46^

*Nimbadon lavarackorum*

Body mass: 70000 g

Justification: Richards et al.^46^

*Litokoala kutjamarpensis*

Body mass: 3700 g

Justification: Black et al.^51^

*Litokoala dicksmithi*

Body mass: 3300 g

Justification: Black et al.^51^

*Madakoala* spp.

Body mass: 9000 g

Justification: Black et al.^51^

*Nimiokoala greystanesi*

Body mass: 3600 g

Justification: Black et al.^51^

*Perikoala robustus*

Body mass: 5100 g

Justification: Black et al.^51^

*Phascolarctos cinereus*

Age: 0 MYA

Justification: extant

Body mass: 6528.74 g

Justification: PanTHERIA database^47^

*Wakaleo pitikantensis*

Body mass: 23300 kg

Justification: *Wakaleo pitikantensis* is very poorly known, making estimating its body mass difficult. Instead, we have used the mean of estimates for the very closely related species, *W. schouteni*, presented by Gillespie et al.^52^

*Priscileo roskellyae*

Body mass: 1813 g

Justification: Gillespie et al.^53^

*Wakaleo oldfieldi*

Body mass: 28400 g

Justification: Wroe et al.^54^

*Wakaleo_vanderleuri*

Body mass: 41400 g

Justification: Wroe et al.^54^

*Sedophascolomys medius*

Body mass: 70000 g

Justification: Murray^11^

*Phascolonus gigas*

Body mass: 599250 g

Justification: Mean of estimates from Richards et al.^46^

*Ramsayia magna*

Body mass: 100000 g

Justification: Johnson and Prideaux^55^

*Lasiorhinus krefftii*

Body mass: 31849.99 g

Justification: PanTHERIA database^47^

*Lasiorhinus latifrons*

Age: 0 MYA

Justification: extant

Body mass: 26163.8 g

Justification: PanTHERIA database^47^

*Mukupirna nambensis*

Body mass: 157999.6 g

Justification: mean of the three postcranial estimates using the regression equation of Richards et al.^46^ —see main text

*Vombatus* *ursinus*

Body mass: 26000 g

Justification: PanTHERIA database^47^

*Vombatus hacketti*

Body mass: 30000 g

Justification: Johnson and Prideaux^55^

*Warendja wakefieldi*

Body mass: 7500 g

Justification: Brewer et al.^10^

*Muramura* spp.

Body mass: 17749.36 g

Justification: Mean of three postcranial equations of Richards et al.^46^ based on humerus and femur circumference measurements for *Muramura williamsi* (pers. obv.).

*Priscakoala lucyturnbullae*

Body mass: 5100 g

Justification: Black et al.^51^

*Zygomaturus trilobus*

Body mass: 128400 g

Justification: Camens^56^

*Diprotodon notatum*

Body mass: 2428400 g

Justification: Mean of the three postcranial equations of Richards et al.^46^ using the mean humeral and femoral circumference measurements of Wroe et al.^57^

*Euryzygoma dunense*

Body mass: 500000 kg

Justification: Sharp^58^

*Propalorchestes novaculacephalus*

Body mass: 155400 g

Justification: Richards et al.’s^46^ estimate for *Propalorchestes* sp.

*Palorchestes painei*

Body mass: 128331.83 g

Justification: mean of mass estimates from the 3UPW, TSL, 4UMA and 4LMA regression equations of Myers^45^ using dental measurements from Woodburne^59^

*Palorchestes azael*

Body mass: 1254700 kg

Justification: mean of estimates of Richards et al.^46^

*Silvabestius spp.*

Body mass: 49627.89 g

Justification: we calculated a mean mass estimate for *Silvabestius johnnilandi* (= 65116.88 g) and a mean mass estimate for S. *michaelbirti* (= 34138.9 g) using the 3UPW, TSL, 4UMA and 4LMA regression equations of Myers^45^, with dental measurements taken from Black and Archer^60^. We then calculated a mean of the estimates for the two species.

*Namilamadeta* spp.

Body mass: 14961.65 g

Justification: we calculated a mean mass estimate for *Namilamadeta* *superior* (= 19467.07 g) using the TSL, 3UPW, 4UMA regression equations of Myers^45^, a mean mass estimate for *N. albivenator* (= 11709.62 g) using the 3UPW, 4UMA, 4LMA regression equations, a mean mass estimate for *N. crassirostrum* (= 14581.15 g) using the 3UPW, 4UMA, 4LMA regression equations, and a mean mass estimate for *N. snideri* (= 14088.74 g) using the 3UPW and 4UMA regression equations, with dental measurements taken from Pledge^2^. We then calculated a mean of the estimates for the four species.

*Ilaria* spp.

Body mass: 154669.02 g

Justification: we calculated a mean mass estimate for *Ilaria illumidens* (= 160068.33 g) using the UMORL, 3UPW and 4UMA regression equations of Myers^45^, and a mean mass estimate for I. lawsoni (= 149269.7 g) using the 4LMA regression equation, with dental measurements taken from Tedford and Woodburne^61^. We then calculated a mean of the estimates for the two species.

*Kuterintja ngama*

Body mass: 16074.57 g

Justification: mean of mass estimates from the 3UPW, 4UMA and 4LMA regression equations of Myers^45^ using dental measurements from Myers and Archer^62^.

Thylacoleo carnifex

Body mass: 57250 g

Justification: mean of estimates of Richards et al.^46^

*Nimbavombatus boodjamullensis*

Body mass: 7500 g

Justification: Brewer et al.^10^

**References**

1 Pledge, N. S. A new species of *Muramura* Pledge (Wynyardiidae: Marsupialia) from the middle Tertiary of the Callabonna Basin, northeastern South Australia. *Bull Am Mus Nat Hist* **279**, 541-555 (2003).

2 Pledge, N. S. The Riversleigh wynyardiids. *Mem Queensl Mus* **51**, 135-169 (2005).

3 Cope, E. D. Synopsis of the Vertebrata of the Puerco Eocene epoch. *Proc Am Philos Soc* **20**, 461-471 (1882).

4 Cope, E. D. The Condylarthra. *The American Naturalist* **18**, 790-805 (1884).

5 Rose, K. D. *The beginning of the age of mammals*. (Johns Hopkins University Press, 2006).

6 Rich, T. H. & Archer, M. *Namilamadeta snideri*, a new diprotodontan (Marsupialia, Vombatoidea) from the medial Miocene of South Australia. *Alcheringa* **3**, 197-208 (1979).

7 Voss, R. S. & Jansa, S. A. Phylogenetic studies on didelphid marsupials II. Nonmolecular data and new IRBP sequences: separate and combined analyses of didelphine relationships with denser taxon sampling. *Bull Am Mus Nat Hist* **276**, 1-82 (2003).

8 Voss, R. S. & Jansa, S. A. Phylogenetic relationships and classification of didelphid marsupials, an extant radiation of New World metatherian mammals. *Bull Am Mus Nat Hist* **322**, 1-177 (2009).

9 Brewer, P. New record of *Warendja wakefieldi* (Vombatidae; Marsupialia) from Wombeyan Caves, New South Wales. *Alcheringa: An Australasian Journal of Palaeontology* **31**, 153-171, doi:10.1080/03115510701305132 (2007).

10 Brewer, P., Archer, M., Hand, S. J. & Abel, R. New genus of primitive wombat (Vombatidae, Marsupialia) from Miocene deposits in the Riversleigh World Heritage Area (Queensland, Australia). *Palaeontol Electron* **8.1.9A**, 1-40 (2015).

11 Murray, P. F. in *Wombats* (eds R. T. Wells & P. A. Pridmore) 1-33 (Surrey Beatty and Sons, 1998).

12 Crompton, A. W. Masticatory motor programs in Australian herbivorous mammals: diprotodontia. *Integr Comp Biol* **51**, 271-281, doi:10.1093/icb/icr028 (2011).

13 Crompton, A. W., Lieberman, D. E., Owerkowicz, T., Baudinette, R. V. & Skinner, J. in *Primate craniofacial function and biology* (eds C. J. Vinyard, M. J. Ravosa, & C. E. Wall) (Springer, 2008).

14 Pledge, N. S. in *Possums and opossums: studies in evolution* (ed Michael Archer) 393-400 (Surrey Beatty & Sons, and the Royal Zoological Society of New South Wales, 1987).

15 Aplin, K. & Archer, M. in *Possums and opossums: studies in evolution.* (ed M. Archer) Ch. 1, xv–lxxii (Surrey Beatty and Sons and the Royal Zoological Society of New South Wales, 1987).

16 Aplin, K. P. in *Possums and opossums: studies in evolution* (ed Michael Archer) Ch. 1, 369-391 (Surrey Beatty & Sons, 1987).

17 Aplin, K. P. *Basicranial regions of diprotodontian marsupials: anatomy, ontogeny and phylogeny*, University of New South Wales, School of Biological Sciences, (1990).

18 Munson, C. J. Postcranial descriptions of *Ilaria* and *Ngapakaldia* (Vombatiformes, Marsupialia) and the phylogeny of the vombatiforms based on postcranial morphology. *Univ Calif Publ Zool* **125** (1992).

19 Barbour, R. A. The musculature and limb plexuses of *Trichosurus vulpecula*. *Aust J Zool* **11**, 488-610 (1963).

20 Argot, C. Functional-adaptive anatomy of the forelimb in the Didelphidae, and the paleobiology of the Paleocene marsupials *Mayulestes ferox* and *Pucadelphys andinus*. *J Morphol* **247**, 51-79, doi:10.1002/1097-4687(200101)247:1<51::Aid-Jmor1003>3.0.Co;2-# (2001).

21 Grand, T. I. & Barboza, P. S. Anatomy and development of the koala, *Phascolarctos cinereus*: an evolutionary perspective on the superfamily Vombatoidea. *Anat Embryol (Berl)* **203**, 211-223 (2001).

22 Black, K. H., Camens, A. B., Archer, M. & Hand, S. J. Herds overhead: *Nimbadon lavarackorum* (Diprotodontidae), heavyweight marsupial herbivores in the Miocene forests of Australia. *PLoS ONE* **7**, e48213, doi:10.1371/journal.pone.0048213 (2012).

23 Hopkins, S. S. B. & Davis, E. B. Quantitative morphological proxies for fossoriality in small mammals. *J Mammal* **90**, 1449-1460 (2009).

24 Weisbecker, V. & Archer, M. Parallel evolution of hand anatomy in kangaroos and vombatiform marsupials: Functional and evolutionary implications. *Palaeontology* **51**, 321-338, doi:10.1111/j.1475-4983.2007.00750.x (2008).

25 Weisbecker, V. & Sánchez-Villagra, M. R. Carpal evolution in diprotodontian marsupials. *Zool J Linn Soc* **146**, 369–384 (2006).

26 Emery, C. in *Zoologische Forschungsreisen in Australien und dem Malayischen Archipel, Vol. 2: Monotremen und Marsupialier* (ed R. Semon) 369-400 (Gustav Fischer, 1897).

27 Flower, W. H. *Osteology of the Mammalia*. (Macmillan, 1867).

28 Scott, G. G. & Richardson, K. C. Appendicular osteological differences between Lasiorhinus latifrons (Owen, 1845) and Vombatus ursinus (Shaw, 1800) (Marsupialia: Vombatidae). *Records of the South Australian Museum* **22**, 95-102 (1988).

29 Jones, F. W. A re-examination of the skeletal characters of *Wynyardia bassiana*, an extinct Tasmanian marsupial. *Pap Proc R Soc Tasman* **1930**, 96-115 (1930).

30 Beck, R. M. D., Warburton, N. M., Archer, M., Hand, S. J. & Aplin, K. P. Going underground: postcranial morphology of the early Miocene marsupial mole *Naraboryctes philcreaseri* and the evolution of fossoriality in notoryctemorphians. *Mem Mus Vic* **74**, 151-171 (2016).

31 Warburton, N. M. *Functional morphology and evolution of marsupial moles (Marsupialia; Notoryctemorphia)*, University of Western Australia, (2003).

32 Warburton, N. M. Functional morphology of marsupial moles (Marsupialia, Notoryctidae). *Verh Natwiss Ver Hambg* **42**, 39-149 (2006).

33 Beck, R. M. D. An 'ameridelphian' marsupial from the early Eocene of Australia supports a complex model of Southern Hemisphere marsupial biogeography. *Naturwissenschaften* **99**, 715-729, doi:10.1007/s00114-012-0953-x (2012).

34 Szalay, F. S. *Evolutionary history of the marsupials and an analysis of osteological characters*. (Cambridge University Press, 1994).

35 Black, K. Maradidae: a new family of vombatomorphian marsupial from the late Oligocene of Riversleigh, northwestern Queensland. *Alcheringa* **31**, 17-32, doi:10.1080/03115510601123601 (2007).

36 Woodhead, J. *et al.* Developing a radiometrically-dated chronologic sequence for Neogene biotic change in Australia, from the Riversleigh World Heritage Area of Queensland. *Gondwana Res* **29**, 153-167, doi:10.1016/j.gr.2014.10.004 (2014).

37 Arena, D. A. *et al.* Mammalian lineages and the biostratigraphy and biochronology of Cenozoic faunas from the Riversleigh World Heritage Area, Australia. *Lethaia* **49**, 43-60 (2015).

38 Trusler, P. W. & Sharp, A. C. Description of new cranial material of *Propalorchestes* (Marsupialia: Palorchestidae) from the middle Miocene Camfield Beds, Northern Territory, Australia. *Mem Mus Vic* **74**, 291-324 (2016).

39 Black, K. Description of new material for *Propalorchestes novaculacephalus* (Marsupialia: Palorchestidae) from the mid Miocene of Riversleigh, northwestern Queensland. *Alcheringa* **30**, 351-361, doi:10.1080/03115510608619322 (2006).

40 Murray, P. Primitive marsupial tapirs (*Propalorchestes novaculacephalus* Murray and *P. ponticulus* sp. nov.) from the mid-Miocene of north Australia (Marsupialia: Palorchestidae). *The Beagle, Records of the Northern Territory Museum of Arts and Sciences* **7**, 39-51 (1990).

41 Murray, P. F. *Propalorchestes novaculacephalus* gen. sp.nov., a new palorchestid (Diprotodontoidea: Marsupialia) from the Middle Miocene Camfield Beds, Northern Territory, Australia. *Tire Beagle, Occasional Papers of tire Nortlrem Territory Museum of Arts and Sciences* **3**, 195-211 (1986).

42 Gillespie, A. K. *Diversity and systematics of marsupial lions from the Riversleigh World Heritage Area and the evolution of the Thylacoleonidae*, University of New South Wales, (2007).

43 Archer, M. in *Vertebrate zoogeography and evolution in Australasia* (eds Michael Archer & Georgina Clayton) 633-808 (Hesperian Press, 1984).

44 Archer, M. & Rich, T. H. in *Carnivorous marsupials* (ed M. Archer) Ch. 2, 495-502 (Royal Zoological Society of New South Wales, 1982).

45 Myers, T. J. Prediction of marsupial body mass. *Aust J Zool* **49**, 99-118 (2001).

46 Richards, H. L., Wells, R. T., Evans, A. R., Fitzgerald, E. M. G. & Adams, J. W. The extraordinary osteology and functional morphology of the limbs in Palorchestidae, a family of strange extinct marsupial giants. *PLoS ONE* **14**, e0221824, doi:10.1371/journal.pone.0221824 (2019).

47 Jones, K. E. *et al.* PanTHERIA: a species-level database of life history, ecology, and geography of extant and recently extinct mammals. *Ecology* **9**, 2648 (2009).

48 Beck, R. M. D. A peculiar faunivorous metatherian from the early Eocene of Australia. *Acta Palaeontol Pol* **60**, 123-129, doi:10.4202/app.2013.0011 (2015).

49 Warburton, N. M. & Travouillon, K. J. The biology and palaeontology of the Peramelemorphia: a review of current knowledge and future research directions. *Aust J Zool* **64**, 151, doi:10.1071/zo16003 (2016).

50 Murray, P. *et al.* Morphology, systematics and evolution of the marsupial genus *Neohelos* Stirton (Diprotodontidae, Zygomaturinae). *Museums and Art Galleries of the Northern Territory Research Report* **6**, 1-141 (2000).

51 Black, K. H., Price, G. J., Archer, M. & Hand, S. J. Bearing up well? Understanding the past, present and future of Australia's koalas. *Gondwana Res* **25**, 1186-1201, doi:10.1016/j.gr.2013.12.008 (2014).

52 Gillespie, A. K., Archer, M. & Hand, S. J. A new Oligo–Miocene marsupial lion from Australia and revision of the family Thylacoleonidae. *J Syst Palaeontol*, 1-31, doi:10.1080/14772019.2017.1391885 (2017).

53 Gillespie, A. K., Archer, M. & Hand, S. J. A tiny new marsupial lion (Marsupialia, Thylacoleonidae) from the early Miocene of Australia. *Palaeontol Electron* **9.2.26A**, 1-26 (2016).

54 Wroe, S., Argot, C. & Dickman, C. On the rarity of big fierce carnivores and primacy of isolation and area: tracking large mammalian carnivore diversity on two isolated continents. *Proceedings. Biological sciences / The Royal Society* **271**, 1203-1211, doi:10.1098/rspb.2004.2694 (2004).

55 Johnson, C. N. & Prideaux, G. J. Extinctions of herbivorous mammals in the late Pleistocene of Australia in relation to their feeding ecology: No evidence for environmental change as cause of extinction. *Austral Ecol* **29**, 553-557 (2004).

56 Camens, A. B. *Systematic and palaeobiological implications of postcranial morphology in the Diprotodontidae (Marsupialia)*, University of Adelaide, (2008).

57 Wroe, S., Crowther, M., Dortch, J. & Chong, J. The size of the largest marsupial and why it matters. *Proceedings. Biological sciences / The Royal Society* **271 Suppl 3**, S34-36, doi:10.1098/rsbl.2003.0095 (2004).

58 Sharp, A. C. A quantitative comparative analysis of the size of the frontoparietal sinuses and brain in vombatiform marsupials. *Mem Mus Vic* **74**, 331-342 (2016).

59 Woodburne, M. O. The Alcoota Fauna, central Australia. *Bulletin of the Bureau of Mineral Resources Geology and Geophysics, Australia* **87**, 1-187 (1967).

60 Black, K. & Archer, M. *Silvabestius* gen. nov. a primitive zygomaturine (Marsupialia, Diprotodontidae) from Riversleigh, northwestern Queensland. *Mem Queensl Mus* **41**, 193-208 (1997).

61 Tedford, R. H. & Woodburne, M. O. in *Possums and opossums: studies in evolution* (ed M. Archer) Ch. 2, 401-418 (Surrey Beatty and the Royal Zoological Society of New South Wales., 1987).

62 Myers, T. J. & Archer, M. *Kuterintja ngama* (Marsupialia, Ilariidae): a revised systematic analysis based on material from the late Oligocene of Riversleigh, northwestern Queensland. *Mem Queensl Mus* **41**, 379-392 (1997).
